# Anharmonic kinetics of the cyclopentane reaction with hydroxyl radical†

**DOI:** 10.1039/c9sc05632g

**Published:** 2020-01-25

**Authors:** Junjun Wu, Lu Gem Gao, Wei Ren, Donald G. Truhlar

**Affiliations:** Department of Mechanical and Automation Engineering, Shenzhen Research Institute, The Chinese University of Hong Kong New Territories Hong Kong SAR China renwei@mae.cuhk.edu.hk; Department of Chemistry, Chemical Theory Center and Supercomputing Institute, University of Minnesota Minneapolis USA truhlar@umn.edu; Center for Combustion Energy, Department of Energy and Power Engineering, Key Laboratory for Thermal Science and Power Engineering of Ministry of Education, Tsinghua University Beijing China

## Abstract

Cyclopentane is one of the major constituents of transportation fuels, especially jet fuel and diesel, and also is a volatile organic compound with a significant presence in the atmosphere. Hydrogen abstraction from cyclopentane by hydroxyl radical plays a significant role in combustion and atmospheric chemistry. In this work we study the kinetics of this reaction at 200–2000 K using direct dynamics calculations in which the potential energy surface is obtained by quantum mechanical electronic structure calculations. The forward and reverse barrier heights and reaction energies obtained by the CCSD(T)-F12a/jun-cc-pVTZ coupled cluster calculations are used as a benchmark to select an accurate electronic structure method among 36 combinations of exchange-correlation functional and basis set. The selected M06-2X/MG3S method shows the best performance with a mean unsigned deviation from the benchmark of only 0.22 kcal mol^−1^ for reaction energies and barrier heights. A quadratic–quartic function is adopted to describe the ring bending potential of cyclopentane, and the quartic anharmonicity in the bending mode is treated by a one-dimensional Schrödinger equation using both Wentzel–Kramers–Brillouin (WKB) and Fourier Grid Hamiltonian (FGH) methods. The torsional anharmonicity in the transition state is treated in turn by the free rotor approximation, the one-dimensional hindered rotor approximation, and the multi-structural torsional anharmonicity method. Rate constants of the title reaction are computed by canonical variational transition state theory including tunneling by the multi-dimensional small-curvature tunneling approximation (CVT/SCT). The final rate constants include the quasiharmonic, quadratic–quartic, and torsional anharmonicity. Our calculations are in excellent agreement with all the experimental data available at both combustion and atmospheric temperatures with a deviation of less than 30%.

## Abstract 2

Quadratic–quartic bend anharmonicity and torsional anharmonicities play important roles in the kinetics of a cyclic hydrocarbon.

## Graphical abstract

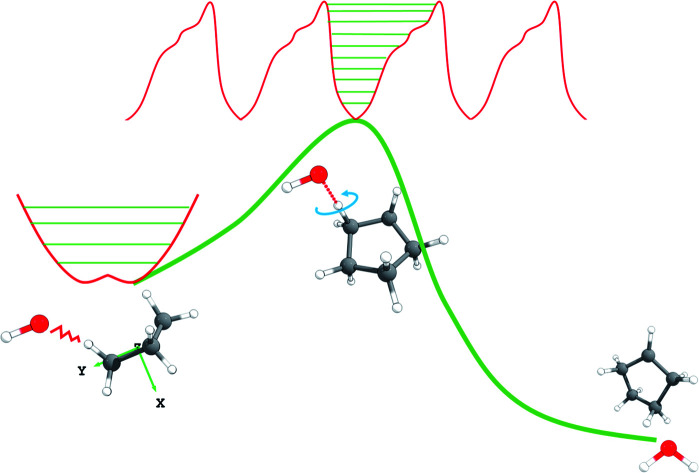

## 1. Introduction

Cycloalkanes are major components in transportation fuels with content of ∼10–15 wt% in gasoline and ∼40 wt% in diesel fuels.^1,2^ Cyclopentane and cyclohexane, due to their abundance in fuel derived from oil sand or shale,^3^ are often selected as prototypes to understand the combustion chemistry of cyclic alkanes. Considerable effort has been dedicated to the development of combustion models for cyclohexane,^4–11^ but the combustion chemistry of cyclopentane, which has the highest octane number among C_5_ to C_8_ cycloalkanes,^2^ is less studied^12,13^ and accurate reaction kinetics are lacking for cyclopentane. Cyclopentane is also a prototype for volatile organic compounds (VOCs) in the atmosphere.^14,15^

Hydrogen abstraction from cycloalkanes has been recognized as the major fuel consumption pathway;^16,17^ however, reliable rate constants of the abstraction from cyclopentane over a wide temperature range are not available. In a recent combustion model of cyclopentane,^12^ the rate constants of many hydrogen abstraction reactions were estimated based on rate rules, which were further tuned to reproduce experimental data on related compounds. Such a treatment may lead to unreasonable rate constants that violate physical limits.^18^ Therefore we embarked on an effort to obtain accurate data over a wide temperature range.

The reaction of cyclopentane with hydroxyl radical (OH) is of particular interest due to its essential role in both combustion chemistry and atmospheric chemistry. The rate constants of the title reaction at atmospheric temperatures have been intensively measured. Jolly *et al.*^19^ measured the rate constant at 298 K using the flash photolysis resonance absorption technique. Several follow-up experiments extended the rate constants to the temperature range of 200–500 K.^20–23^ Most of the previous measurements were conducted based on the relative rate method, but recently Gennaco *et al.*^24^ used laser-induced fluorescence to directly measure the rate constants at 233–351 K. Good consistency is found among these lower-temperature measurements. At combustion temperatures, Sivaramakrishnan *et al.*^25^ measured the rate constants of cyclopentane with OH at 801–1347 K in shock tube experiments.

It would be useful to have a high-accuracy theoretical rate constant determination to extend the range of these measurements, but this is not available. Theoretical rate constant calculations are important not only because they can extend experiments to other temperatures, but also because if a method is validated for cyclopentane, it can be used to predict accurate results for substituted cyclopentanes where experimental data are unavailable. To date, only two theoretical studies have been conducted to predict the rate constants.^25,26^ Sivaramakrishnan and Michael used conventional transition state theory (TST) to calculate the rate constants with electronic structure calculations carried out by G3//B3LYP/6-31G(d).^25^ The torsional anharmonicity was treated by the free-rotor assumption and the tunneling effect was accounted by the Wigner correction using a scaled imaginary frequency. This work reproduced the experimental data, but possibly due to error cancellation since the methods are not expected to be generally reliable,^27^ but such cancellation cannot be relied upon for substituted cycloalkanes. In the other work, Yu and Zhang used canonical variational transition state theory and small-curvature tunneling (CVT/SCT) with a low-level reaction path and selected energies calculated at the CCSD(T)/aug-cc-pVTZ level to determine rate constants from 200 to 900 K.^26^ The CVT method includes the variational effect (recrossing of the conventional transition state dividing surface) that plays an important role in many radical-tunneling reactions, and the SCT approximation is much more reliable than the low-order Wigner method. Although the method of calculation is greatly improved, this work underestimated the experimental data, apparently due to neglect of anharmonicity. Another concern is that the previous theoretical work ignored the conformations of cyclopentane. The ring-puckering of cyclopentane gives two conformations,^28,29^ in particular an envelope and a half-chair. The existence of two conformations is an anharmonic effect since a harmonic potential has only one minimum.

The present work also uses CVT/SCT, but it improves on previous theoretical work in four important ways.

First, we consider a wider temperature range: 200 to 2000 K.

Second, we select an accurate and more efficient electronic structure method to calculate the reaction path and all needed data about the potential energy surface (PES). The optimum choice is made by comparing Kohn–Sham density functional theory (KS-DFT) with a selection of exchange-correlation functionals to a coupled cluster benchmark in order to find a reaction-specific choice of functional that gives good results for the present reaction. The selected best-performance KS-DFT method would, we assume, also be a good choice for other cycloalkanes while remaining affordable (due to the efficiency of KS-DFT) even for larger reactive systems for which well-converged coupled cluster calculations may become prohibitively expensive or even impossible.

**Fig. 1:**
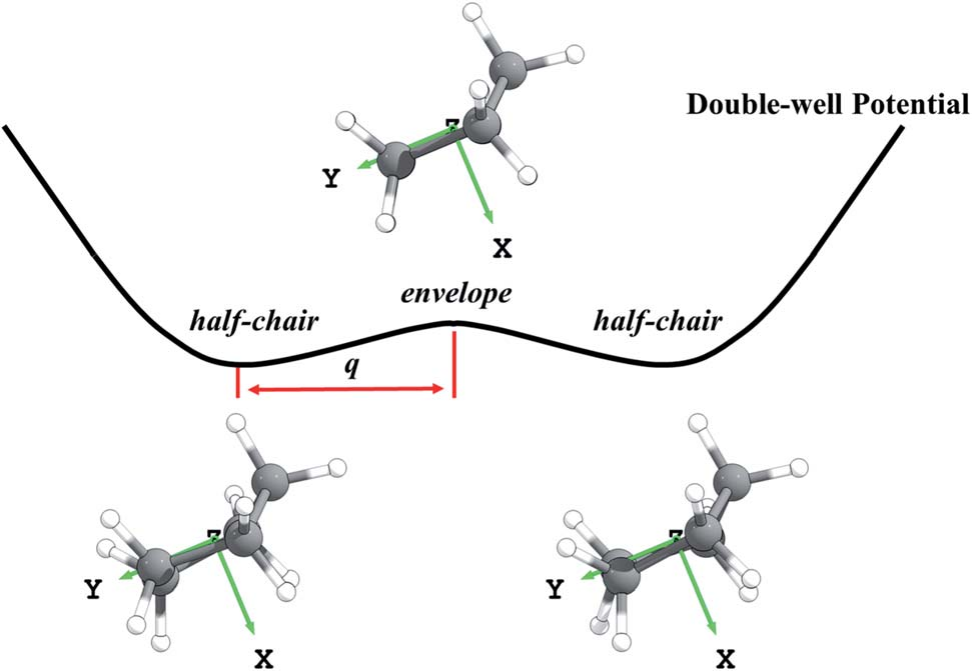
Schematic diagram of the symmetric double-well potential of the bending mode in cyclopentane. The envelope conformation is a saddle point that connects the half-chair mirror structures; *q* denotes the distance between the half-chair and the envelope structures.

Third, we consider the quartic anharmonicity of cyclopentane. The envelope and half-chair conformations are interconverted by a low-frequency bending mode; however, we identify the envelope conformation as the saddle point that connects one half-chair conformer to its mirror image; see Fig. 1. This kind of situation is most readily treated as quartic anharmonicity rather than a conformational effect.^30^ We adopt a quadratic–quartic function to describe the potential connecting these structures, and we obtain the energy levels by solving the one-dimensional Schrödinger equation using Wentzel–Kramers–Brillouin (WKB) and Fourier grid Hamiltonian (FGH) methods. The quartic anharmonicity is incorporated into the final rate constant determination.

Finally, we examine the torsional anharmonicity present at the transition state. Unlike the abstraction from open chains, the cyclic reactant is free of low-frequency torsions, but the newly forming bond creates an internal rotation at the transition state. For hydrogen abstractions by OH, the literature contains three principal approaches to treating the torsional anharmonicity created by the newly formed bond: (i) Ignore the anharmonicity, *i.e.*, use the quasiharmonic treatment for the C–O torsion. (ii) Treat the C–O torsion as an uncoupled hindered rotor or rotors. (iii) Treat the C–O torsion as a coupled hindered rotor. We compare them here for the case where the abstraction is from a hydrogen (H) attached to a ring carbon atom (C). We will compare these treatments, which to our knowledge has not been done before.

## 2. Methodology

### 2.1 Electronic structure determination

First the M08-HX^31^ density functional with the MG3S^32^ basis set was used to optimize the geometries of reactants, transition states, and products. Note that the MG3S basis set is identical to 6-311+G(2df,2p)^33^ for C, H, O and atoms. The M08-HX/MG3S method is chosen for this step based on its capability of predicting accurate internuclear distances at transition states.^34^ Then the explicitly correlated CCSD(T)-F12 (ref. 35) approximation with the jun-cc-pVTZ^36^ basis set was used for single-point energy calculations at these optimized geometries. This method is more accurate and efficient than CCSD(T)/aug-cc-pVTZ or CCSD(T)/aug-cc-pV5Z calculations.^37^ We used the energies predicted by CCSD(T)-F12a^35^/jun-cc-pVTZ^36^//M08-HX^31^/MG3S^32^ as the benchmark to calculate the forward and reverse barrier heights and the energy of reaction. The barrier heights and energy of reaction are critical parameters for kinetics that are used to select the best-performing KS-DFT method. Nine KS-DFT exchange-correlation functionals (B3LYP,^38^ M05-2X,^39^ M06-2X,^40^ M08-HX,^31^ M08-SO,^31^ MN15,^41^ MN15-L,^42^ MP1WK^43^ and ωB97X-D^44^) with four basis sets (MG3S,^32^ jun-cc-pVTZ,^36^ jul-cc-pVTZ^36^ and aug-cc-pVTZ^36^) were tested based on the M08-HX/MG3S geometries. This leads to 36 electronic structure methods, and the one with the smallest mean unsigned error (averaged over the forward and reverse barrier heights and the energy of reaction) is selected for dynamics calculations.

In the search for the envelope and half-chair structures, we found that the geometries and frequencies obtained for the structures are very sensitive to grid size. The two structures initially optimized with ultrafine and superfine grid appeared as minima. A denser grid with 96 shells and 32 *θ* points and 64 *φ* points in each shell was adopted to refine the structures, and this identified the envelope conformer as the saddle point and the half-chairs as minima. The envelope (C_s_) is a saddle point that connects a half-chair (C_2_) to the mirror structure of that half-chair, as shown in Fig. 1. It indicates this bending mode has a symmetric double-well potential.

All the KS-DFT calculations were carried out using the Gaussian 16 program^45^ except for the M08-SO calculations, which were carried out using a locally modified Gaussian 09 program.^46^ An ultrafine grid, which has 99 radial shells and 590 angular points per shell, was used for the KS-DFT calculations. The coupled cluster calculations were performed using the Molpro 2012 program.^47^ The optimized geometries are provided in the ESI.†

### 2.2 Reaction kinetics

The rate constants of the title reaction were calculated at the low-pressure limit by assuming no collisional stabilization of the pre-reactive complex. Both conventional TST and CVT calculations were conducted, where CVT locates the transition state dividing surface at the position along the reaction path that maximizes the free energy of activation.^48^ The quasiclassical CVT rate constant of the bimolecular reaction at temperature *T* is then expressed as:1
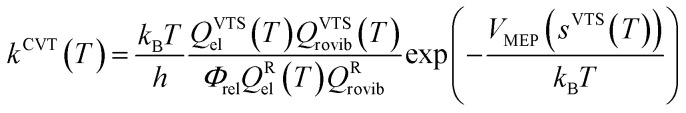
where *k*_B_ is Boltzmann's constant, *h* is Plank's constant, *T* is temperature, *Q*^VTS^_el_(*T*) and *Q*^VTS^_rovib_(*T*) are respectively the electronic partition function and the rotation-vibrational partition function of the variational transition state (VTS); *Q*^R^_el_(*T*) and *Q*^R^_rovib_(*T*) are the electronic and rotation-vibrational partition functions of reactants, respectively; *Φ*_rel_ is the relative translational partition function per unit volume, and *V*_MEP_(*s*) is the potential energy along the isoinertial minimum energy path (MEP) at position *s*, and *s*^VTS^(*T*) is the location of the VTS. Eqn (1) reduces to conventional TST when all transition state evaluations are done at *s* = 0 rather than *s*^VTS^(*T*). Reactant partition functions are computed for the half-chair structure and have their zero of energy at the potential energy of the equilibrium half-chair. The partition functions for the VTS and the conventional transition state (which passes through the saddle point) are on the MEP and at the saddle point, respectively.

The quasiclassical approximation treats the reaction coordinate as separable and treats all vibrational modes except the reaction coordinate motion as quantized. Nonseparability of the reaction coordinate and quantum mechanical effects on the reaction coordinate motion are included by the transmission coefficient *κ*(*T*), which includes both the tunneling and nonclassical reflection. In the present work, *κ*(*T*) is evaluated by two methods for comparison: the multi-dimensional zero-curvature tunneling (ZCT)^49,50^ approximation and the small-curvature tunneling (SCT)^51^ approximation. Thus the rate constant is given by:

2 *k*^CVT/XCT^(*T*) = *κ*(*T*)*k*^CVT^

where XCT stands for ZCT or SCT. The ZCT and SCT approximations differ in that the former neglect reaction-path curvature and the latter includes corner cutting due to reaction-path curvature, but both approximations take the effective potential for tunneling to be the ground-state vibrationally adiabatic potential given by^50^

3 *V*^G^_a_(*s*) = *V*_MEP_(*s*) + *ε*^G^(*s*)

where *ε*^G^(*s*) is the zero point energy in modes normal to the MEP.

The partition functions *Q*^R^_rovib_ and *Q*^VTS^_rovib_, and *Q*^CTS^_rovib_ in eqn (1) and (2) can be written as a product of a rotational partition function *Q*^X^_rot_ and a vibrational partition function *Q*^X^_vib_ (with X = R, VTS, or conventional transition state), and the vibrational partition function is a product of factors corresponding to individual modes. All the vibrational partition functions include the quasiharmonic anharmonicity as explained in Section 2.3. The vibrational partition function factor for the lowest-frequency mode of cyclopentane is replaced by a quadratic–quartic partition function as explained in Section 2.4.

Finally, we include torsional anharmonicity (Anh) of the transition state, and the final rate constant is given by

4 *k*^Anh–CVT/XCT^_τ_(*T*) = *F*^Anh^_τ_*κ*(*T*)*k*^CVT^

where *F*^Anh^_τ_ is explained in Section 2.5.

The direct dynamics calculations were carried out by using the Pilgrim program.^52^ The reaction path, scaled to 1 amu, was computed from −1.0 Å to +1.0 Å by the Page–McIver algorithm^53^ with a step size of 0.005 Å; Hessians were computed every ten steps. The integrations needed for the ZCT and SCT transmission coefficients were converged to 0.1%.

### 2.3 Quasiharmonic anharmonicity

The first step in treating the vibrations is to scale the directly calculated vibrational frequencies. We define the quasiharmonic (QH) approximation as using the harmonic oscillator formulas with scaled vibrational frequencies^54^ parametrized to approximate the anharmonic zero point energy (which is dominated by high-frequency vibrations). In the present case the scaling factor determined by the method of ref. 54 is 0.975. All of our vibrational partition functions are computed using these scaled frequencies.

### 2.4 Quartic anharmonicity

We use the quadratic–quartic formula to describe the double-well potential of the lowest-energy vibrational mode of the envelope structure:5
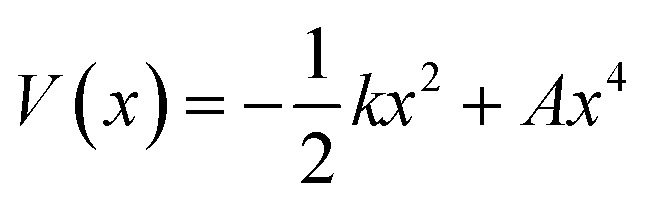
where *k* is the quadratic force constant, *A* is the quartic force constant, and *x* is a bend coordinate with units of length in isoinertial coordinates scaled to a reduced mass *μ* of 1 amu.

Here we summarize the key steps to obtain the bend coordinate and the quadratic–quartic potential; full details are included in the ESI.† In eqn (5), *k* is directly determined by the imaginary frequency *ω* of the envelope conformation at *x* = 0:

6 *k* = −*μω*^2^

Let *q* be the distance between the envelope and half-chair structures on the isoinertial MEP; then *A* is determined by:7
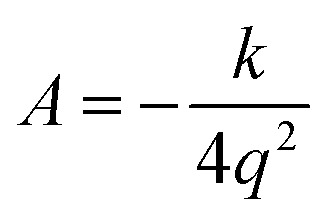


In order to calculate the distance *q*, it is necessary to make the origin and orientation consistent between the two structures. We did this using Chen's method.^55^

Now that the quadratic–quartic potential is available, the next step is to find the eigenvalues *ε*_*i*_ of the one-dimensional Schrödinger equation:8
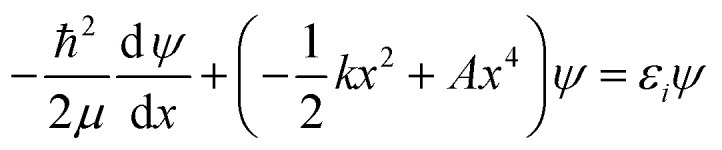


As a check, we did this in two ways, first using the WKB method^56^ and then using the FGH method.^57^ The partition function is computed by:9
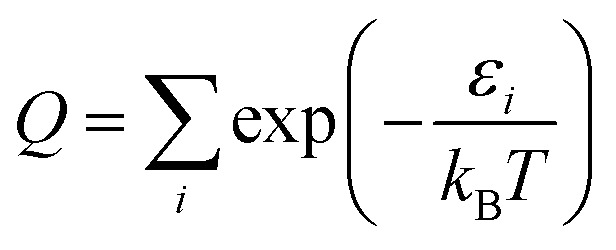


### 2.5 Torsional anharmonicity

Cyclopentane and its products are free of low-frequency torsional modes, but the transition state introduces a torsional motion associated with the abstraction site, and the resulting anharmonicity must be treated accurately to obtain reliable results, especially at high temperatures. A standard method in the literature is to treat torsions as independent and add anharmonicity to individual normal modes corresponding to torsions. A problem with this approach is that low-frequency modes often involve a combination of bends and torsions, and a torsion is often spread out over two or more normal modes. An alternative approach^58,59^ constructs vectors representing torsion motions about given bonds, from which a projection matrix is formed. This projection matrix is applied to the Hessian matrix (along with usual translation and rotation projections) to project out any components of the torsional motions. This means that even though a torsion might be spread across several eigenvectors of the original Hessian, the projection operator will remove the torsion component from any that have it. Once the projection has been applied to the Hessian, it is diagonalized. The frequencies obtained are not necessarily the ones that would have been obtained from diagonalizing the original Hessian matrix without the torsion projection, and therefore the method, like the MS-T method, does not identify a torsion with a single normal mode. This is an improvement, but the method still does not include the coupling of torsions to one another and to overall rotation, as is included in the MS-T method, and this has been shown to led to an overestimation of the entropy in alkanes.^60^

**Fig. 2:**
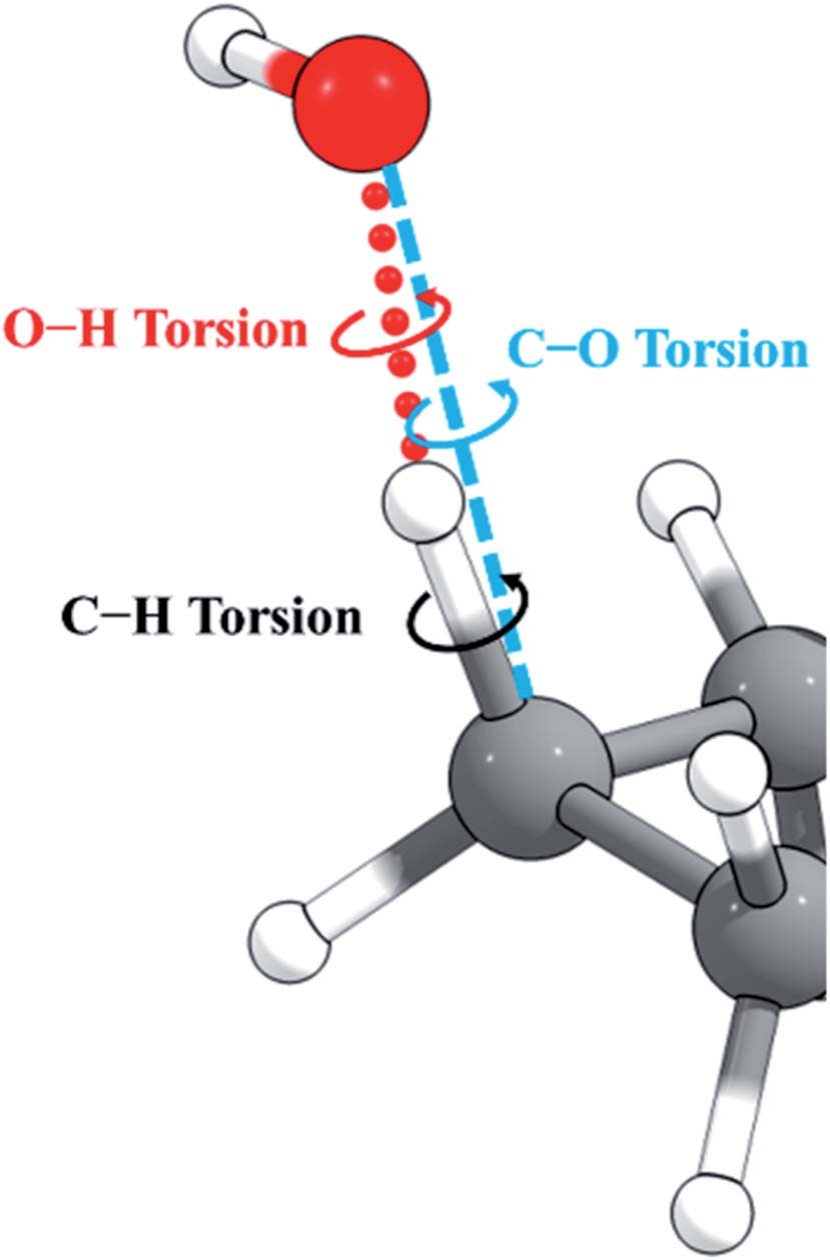
The active site for abstraction of H from a C–H bond by OH. The O–H–C bond angle is bent.

For torsions arising at the site of a hydrogen abstraction by OH or a polyatomic radical, there is a second problem with the independent torsion approximations. This is illustrated in Fig. 2. Because the O–H–C bond angle is not 180°, there are two torsions, one about the forming O–H bond and one about the breaking C–H bond. However, we found that one can get nonphysical results by treating these torsions as independent when the bond angle is close to 180°, as it is in the present case. One can, however, get more reasonable results if one treats this as a single torsion about the C–O axis (which would strictly be the case if O, H, and C were collinear). Therefore, in the present work, we adopt the single-torsion model for the independent torsion approximations. A detailed definition of the internal coordinates for the two scenarios is given in the ESI.†

Here we investigate the anharmonicity of the transition state by three methods, in particular two independent-torsion methods, namely the free rotor (FR) approximation and a hindered rotation (HR) method,^61^ and one coupled-torsion method, namely the multi-structural torsion (MS-T) method.^62^ Next we recall the essentials of these three methods. For both the CVT calculation and conventional TST calculation, torsional anharmonicity was estimated at the conventional transition state, not at the VTS.

#### Free rotation (FR)

At very high temperature and/or for a low torsional barrier height, in particular when *k*_B_*T* ≫ *V*_barrier_, the anharmonic partition function becomes free internal rotation. The FR approximation uses this approximation at all *T* and replaces the quasiharmonic approximation for one normal mode by10
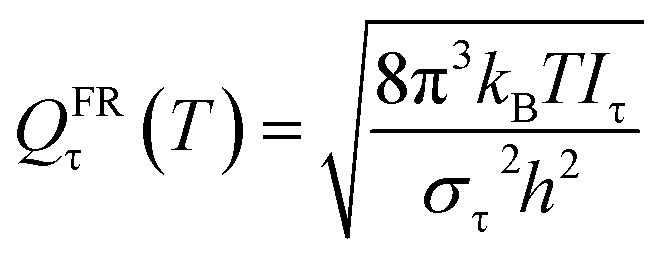
where τ labels the torsion, *T* is the temperature*, σ*_τ_ is the symmetry number, which is 1 in this work, and *I*_τ_ is torsional moment of inertia, which is calculated to be 1.51 amu Å^2^ in this work.

#### Hindered rotation (HR)

For an HR approximation to be accurate at all temperatures, it should interpolate the partition function smoothly between the high-temperature region where the FR approximation is a reasonable zero-order model to the low-temperature regime where the QH approximation is a reasonable zero-order model. A variety of inexpensive treatments are available along this line.^63,64^ Here we use the torsional eigenvalue summation (TES) method since it can provide an accurate treatment of a one-dimensional separable torsion.^64^ In these calculations, the one-dimensional torsional potential (*V*) as a function of the torsion angle (*ϕ*) is represented by a Fourier series:11
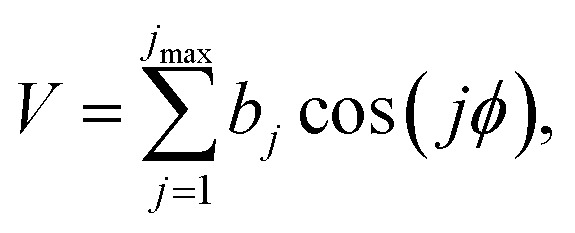
where *j*_max_ is the maximum order (taken here as 10), and *b*_*j*_ is a coefficient. We calculate the spectrum of eigenvalues of the 1D torsional Schrödinger equation to high enough energy to converge the partition function. We then obtain *Q*^HR^_τ_ by summation as in eqn (9), and we substitute it for the quasiharmonic approximation of the mode selected to be treated as a torsion.

We applied eqn (11) only at the saddle point, and we assumed that the anharmonicity factor calculated at the saddle point can also be used along the reaction path. This approximation is similar to the approximation in the original MS-CVT/SCT method,^65^ in which the multistructural anharmonicity factors are based on stationary points; in contrast the MS(full)-CVT/SCT method denotes the calculation of multiple structures along the reaction path. In previous work, Bao *et al.*^66^ explored the difference between the MS-CVT/SCT and MS(full)-CVT/SCT treatments of the kinetics. In the case studied, it was found these two treatments give almost the identical rate constants over the 200–3000 K temperature range.

#### Multistructural (MS) anharmonicity

In contrast to the above treatments, the MS-T method treats the torsions in internal coordinates in such a way that one does not have to identify torsions with normal modes; it includes torsional anharmonicity and the coupling of torsions to overall rotation (and – when there is more than one torsion – the coupling of torsions to one another); the multiple minima due to the torsion or torsions are treated as multiple conformers.^67^ Only the geometries and Hessians of the conformers are needed in the MS-T method, so it eliminates the tedious torsional potential scan (nor does one have to find torsional barrier heights). The MS-T method calculates the conformational-rotational-vibrational partition function as:^68^12
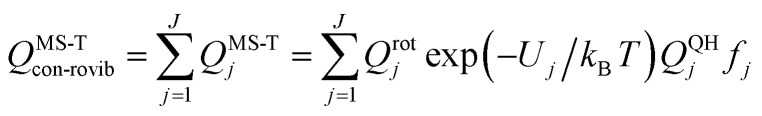
where *Q*^MS-T^_*j*_ is the contribution of conformer *j* to the total MS-T partition function, *Q*^rot^_*j*_ and *Q*^QH^_*j*_ are respectively the rigid rotational and quasiharmonic vibrational partition functions of conformer *j*, *U*_*j*_ is the energy of conformer *j*, and *f*_*j*_ is a factor that accounts for the torsional anharmonicity.

A slightly simpler approximation is to include all the conformers but treat them as locally harmonic (or quasiharmonic). This treatment is labeled MS(LH), and the MS-T partition function reduces to this if we approximate *F*^Anh^_tor_ as 1.

#### Torsional anharmonicity factors

To quantify the torsional anharmonic effect, we define the torsional anharmonicity factor, *F*^Anh^_tor_, as the ratio of the anharmonic conformational-vibrational-rotational partition function to the quasiharmonic (QH) vibrational-rotational partition function. The FR and HR torsional anharmonicity factors are defined as:13
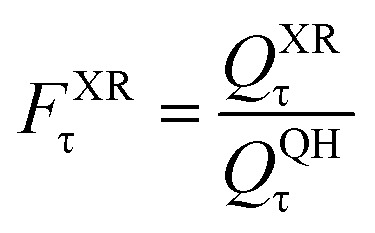
where XR stands for FR or HR, and *Q*^QH^_τ_ is the quasiharmonic vibrational partition function of mode of the transition state that is treated as a torsion.

The MS-T torsional anharmonicity factor is defined as14
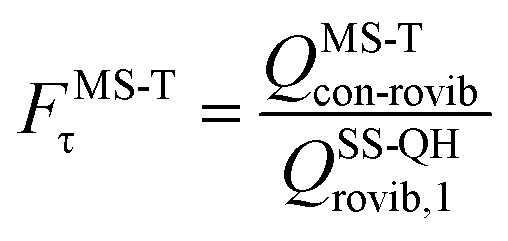
where *Q*^SS-QH^_rovib,1_ is the rigid rotator–quasiharmonic partition function of the lowest-energy conformer. We also consider the MS(LH) method, for which the torsional anharmonicity factor is:15
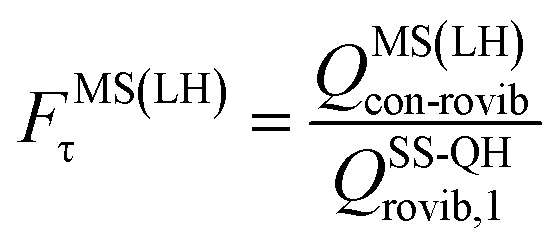


#### Computational details

**Fig. 3:**
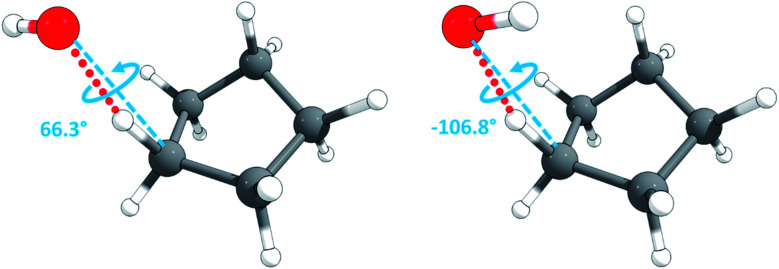
Torsional conformers of the transition state of cyclopentane + OH reaction.

The torsional moment of inertia was calculated by the method of Kilpatrick and Pitzer^69^ using the *kpmoment.exe* utility in the MSTor program suite.^70,71^ The HR partition function was calculated by the TES method^64^ using the *tes.exe* utility, also in the MSTor program suite. The scan for the HR calculation is a relaxed scan of the C–O torsion from 0° to 360° with a spacing of 10°; for each fixed torsional angle, the geometry is optimized to a saddle point (the resulting torsional potential is shown in Fig. S3 of the ESI†). The torsional conformer search was conducted using the *confgen.exe* utility in the MSTor program suite, and two torsional conformers were found for the chair conformation; they are shown in Fig. 3. The MS-T and MS(LH) partition functions were calculated using MSTor program.

## 3. Results and discussion

The half-chair cyclopentane has ten abstraction sites, but optimization of the ten possible transition structures gives an identical twisted geometry, although the ten H-atoms in cyclopentane are not rigorously identical. This is also consistent with the fact that the ten individual C–H BDEs are all equal to 102.25 kcal mol^−1^ (see Table S1 in the ESI†).

**Fig. 4:**
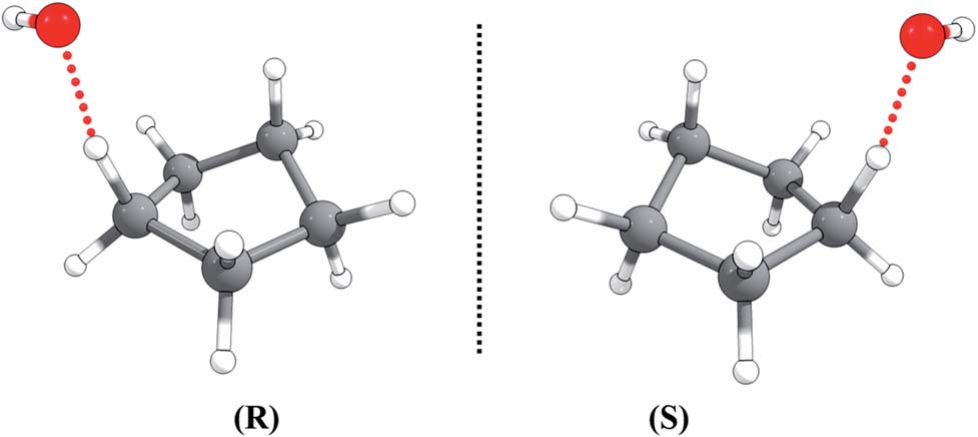
The *rectus* (*R*) and *sinister* (*S*) enantiomers of the transition state for hydrogen abstraction of cyclopentane by OH radical.

The transition state is chiral with C_1_ symmetry; as shown in Fig. 4, it has two enantiomers, *rectus* (*R*) and *sinister* (*S*), *i.e.*, *R*-TS and *S*-TS. The thermal reaction rate constants of the two cyclopentane enantiomers with the non-chiral OH radical are the same, so we only need to consider one enantiomer for rate constant calculations, and the rate constant for that transition enantiomer is multiplied by a factor of 2 to include the other optical isomer.

### 3.1 Selection of electronic structure method

In order to select a method for benchmarking of approximate density functionals, we first consider the *T*_1_ diagnostic. The standard criterion^72^ is that the single-reference CCSD(T) method should be reliable if the *T*_1_ diagnostic is smaller than 0.02 for closed-shell systems and 0.045 for open-shell systems; these criteria are satisfied in the present work. Therefore, we use CCSD(T)-F12a/jun-cc-pVTZ//M08-HX/MG3S to provide benchmarks to choose the KS-DFT method.

We tested nine exchange-correlation functionals (see Section 2.1), and full results are in the ESI.†Table 1 gives results for the two best-performing functionals, each with four basis sets at two sets of geometries for each set of geometries the results are compared to the benchmark calculations at the same geometry. The table shows that the M05-2X and M06-2X density functionals perform quite well, with all MUDs in the range 0.22–0.60 kcal mol^−1^. The M06-2X/MG3S functional shows the best performance, and it is selected for use in the anharmonicity and direct dynamics calculations.

**Fig. 5:**
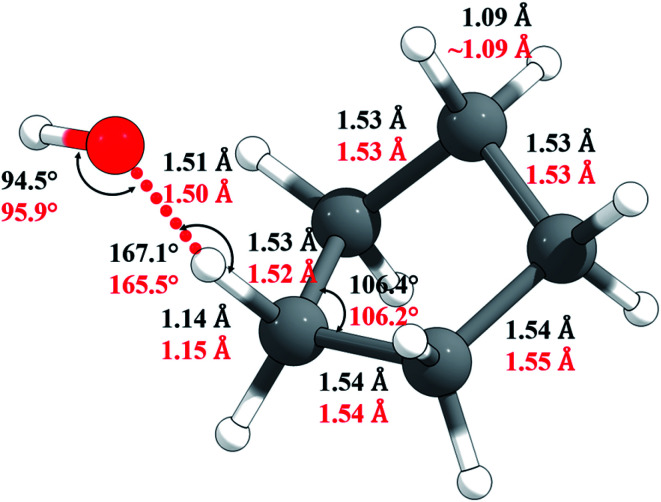
Selected geometric parameters of the transition state optimized by M06-2X/MG3S (in black) and by M08-HX/MG3S (in red).

The M06-2X/MG3S Cartesian coordinates of reactants, products, and transition structure are in the ESI;† key M06-2X/MG3S and M08-HX/MG3S internal coordinates of the transition structure are shown in Fig. 5. The C–H and C–C bond lengths and C–C–C angles in cyclopentane, cyclopentyl, and the transition structure are almost identical between the M06-2X/MG3S and M08-HX/MG3S structures, while the C–H–O and H–O–H angles in the transition structure optimized by M06-2X/MG3S and M08-HX/MG3S differ by 1.4–1.6°. The C–H–O–H dihedral angle is 94.3° by M06-2X/MG3S, and 85.4° by M08-HX. The similarities of these geometries explain why we draw similar conclusions from the upper and lower portions of Table 1.

**Table 1:** The forward and reverse barrier heights (
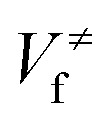
 and 
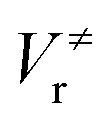
), energies of reaction (Δ*V*), and mean unsigned deviations (MUDs) from the benchmark for the hydrogen abstraction from cyclopentane by OH radical. The single-point energies are calculated based on the geometries optimized by M08-HX/MG3S (upper panel) and M06-2X/MG3S (lower panel). All the energies are relative to the total energies of the reactants in kcal mol^−1^ with ZPE excluded

Method	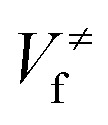	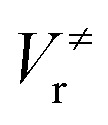	Δ*V*	MUD
**Calculated at geometries optimized by M08-HX/MG3S**				
CCSD(T)-F12a/jun-cc-pVTZ	1.27	22.65	−21.38	—
M05-2X/MG3S	1.05	22.96	−21.91	0.35
M05-2X/jun-cc-pVTZ	0.94	23.16	−22.22	0.56
M05-2X/jul-cc-pVTZ	0.90	23.12	−22.22	0.56
M05-2X/aug-cc-pVTZ	0.83	23.11	−22.27	0.60
**M06-2X/MG3S**	**0.87**	**22.35**	**−21.48**	**0.27**
M06-2X/jun-cc-pVTZ	0.85	22.68	−21.83	0.30
M06-2X/jul-cc-pVTZ	0.80	22.63	−21.83	0.31
M06-2X/aug-cc-pVTZ	0.75	22.62	−21.87	0.35

**Calculated at geometries optimized by M06-2X/MG3S**				
CCSD(T)-F12a/jun-cc-pVTZ	1.18	22.59	−21.40	—
M05-2X/MG3S	1.03	22.90	−21.87	0.31
M05-2X/jun-cc-pVTZ	0.94	23.12	−22.18	0.52
M05-2X/jul-cc-pVTZ	0.89	23.08	−22.19	0.52
M05-2X/aug-cc-pVTZ	0.83	23.07	−22.24	0.56
**M06-2X/MG3S**	**0.85**	**22.32**	**−21.47**	**0.22**
M06-2X/jun-cc-pVTZ	0.84	22.66	−21.82	0.28
M06-2X/jul-cc-pVTZ	0.80	22.61	−21.82	0.27
M06-2X/aug-cc-pVTZ	0.74	22.60	−21.86	0.30

### 3.2 Quartic anharmonicity

The M06-2X/MG3S energies of the *envelope* and *half-chair* structures differ by only 0.8 cal mol^−1^. The frequencies of the bending mode of *envelope* and *half-chair* structures are 10.2*i* and 14.3 cm^−1^, respectively. The second-order force constant *k* is calculated to be −1.52 × 10^−4^ J m^−2^ and the fourth-order force constant *A* is 6.58 × 10^15^ J m^−4^. In fact, we found that the quadratic term is negligible and the potential is almost quartic.

**Fig. 6:**
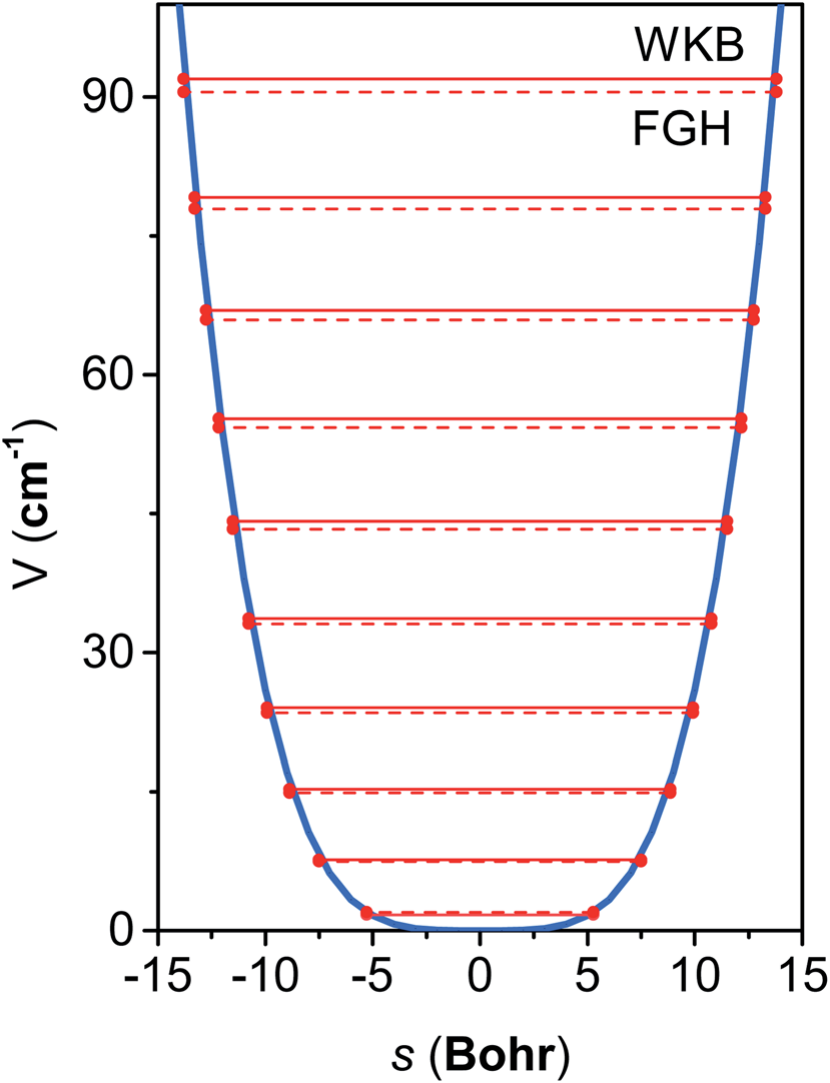
Quadratic–quartic potential along the mass-scaled coordinate for the bending mode in cyclopentane. The top ten eigenvalue spectrum of the potential computed by WKB method (solid line) and FGH method (dash line) is also displaced.

**Fig. 7:**
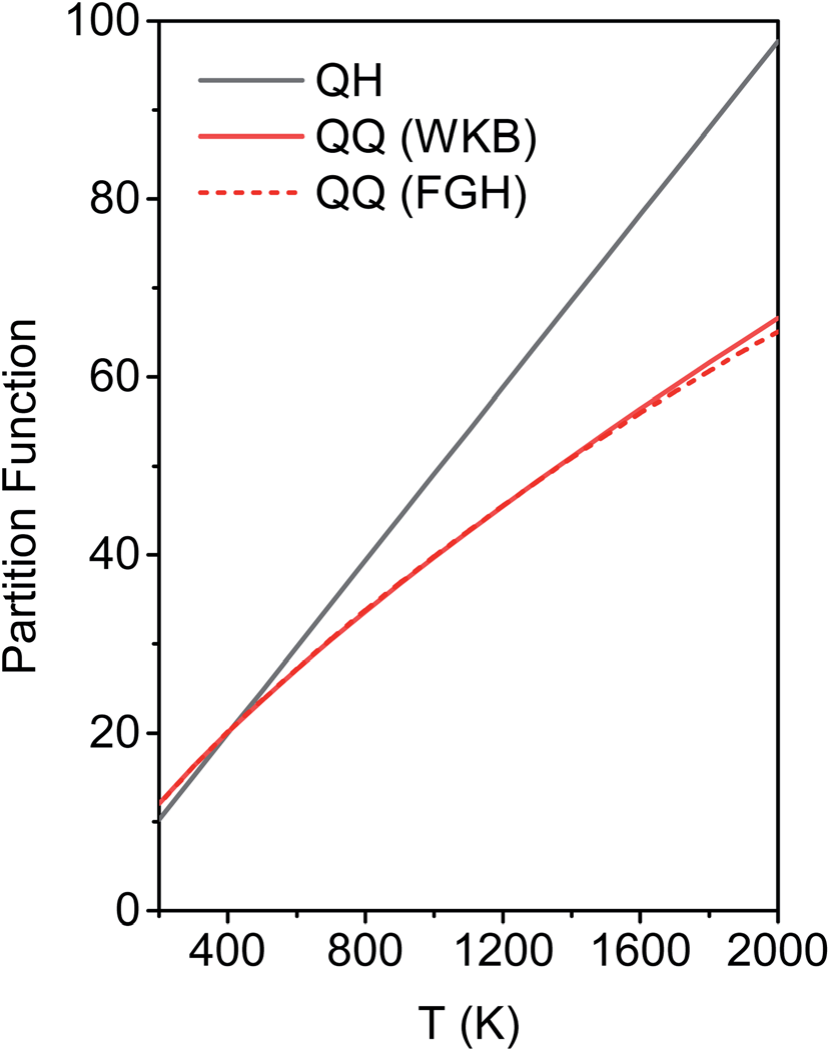
Anharmonic and harmonic partition functions at 200–2000 K. QH is the quasiharmonic oscillator partition function. QQ (WKB) and QQ (FGH) are the quadratic–quartic anharmonic partition functions obtained from the energy levels by WKB method (red solid line) and FGH method (red dashed line), respectively.

Fig. 6 shows the first 10 energy levels calculated by the WKB and FGH methods using the quadratic–quartic potential. It is seen that WKB and FGH methods give very close and consistent energy levels. We calculated enough levels to converge the partition functions to 0.1%. Fig. 7 compares the anharmonic partition functions by WKB and FGH methods and the QH partition function. It is seen that the anharmonic partition functions by WKB and FGH are almost identical, and their ratio to the QH partition function varies from 1.2 at 200 K to 0.7 at 2000 K. This anharmonicity lowers the rate constant by a factor of 0.8 at 200 K and raises the rate constant by a factor of 1.5 at 2000 K.

### 3.3 Torsional anharmonicity

**Fig. 8:**
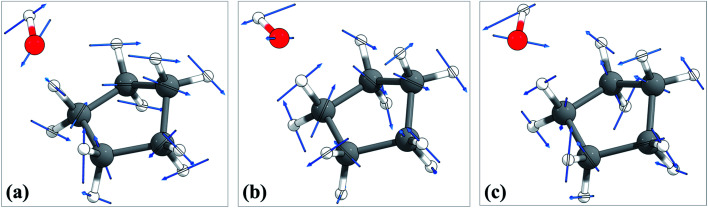
Displacement vectors of three normal modes with frequencies of (a) 66.6 cm^−1^, (b) 76.7 cm^−1^ and (c) 109.1 cm^−1^, which are likely the internal rotations.

The torsional anharmonicity of the transition state is examined by the FR, HR, MS-T and MS-LH methods and compared to the single-structure quasiharmonic treatment. The FR and HR treatments necessitate the manual assignment of the torsion to a specific normal mode. In this work, the torsion is spread out over three low-frequency normal modes, as seen in the normal-mode displacement vectors shown in Fig. 8. The frequencies of the three normal modes are *ω*_1_ = 66.6 cm^−1^, *ω*_2_ = 78.7 cm^−1^, and *ω*_3_ = 109.1 cm^−1^. For simplicity, we use the notation *ω*_1_, *ω*_2_, and *ω*_3_ to represent these low-frequency normal modes.

**Fig. 9:**
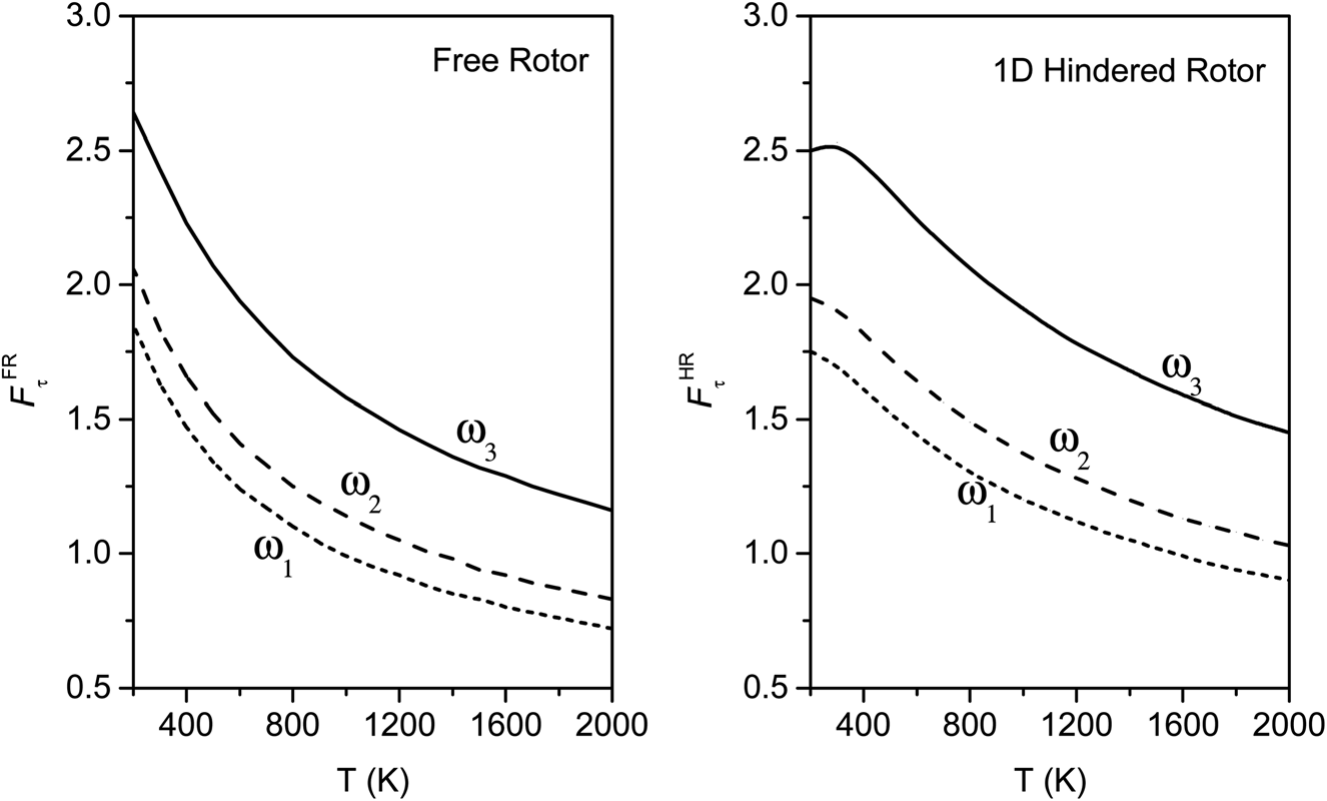
Torsional anharmonicity factors for FR and HR treatments by different assignments of the normal modes to the torsion; *ω*_1_, *ω*_2_ and *ω*_3_ denote the normal modes of frequencies 66.6, 78.7, and 109.1 cm^−1^, respectively. Each curve is labeled by the vibrational mode for which the QH approximation is replaced by the torsional partition function.

We calculated the FR and HR torsional anharmonicity factors with each of the three possible choices of a normal mode to be treated as the torsion. Fig. 9 shows the torsional anharmonicity factors by FR and HR treatments at 200–2000 K. In either the FR or the HR treatment, the torsional anharmonicity factors vary by a factor of ∼1.5 due to different possible assignments of the normal modes to the torsion, which is a severe disadvantage of the independent-torsion methods.

**Fig. 10:**
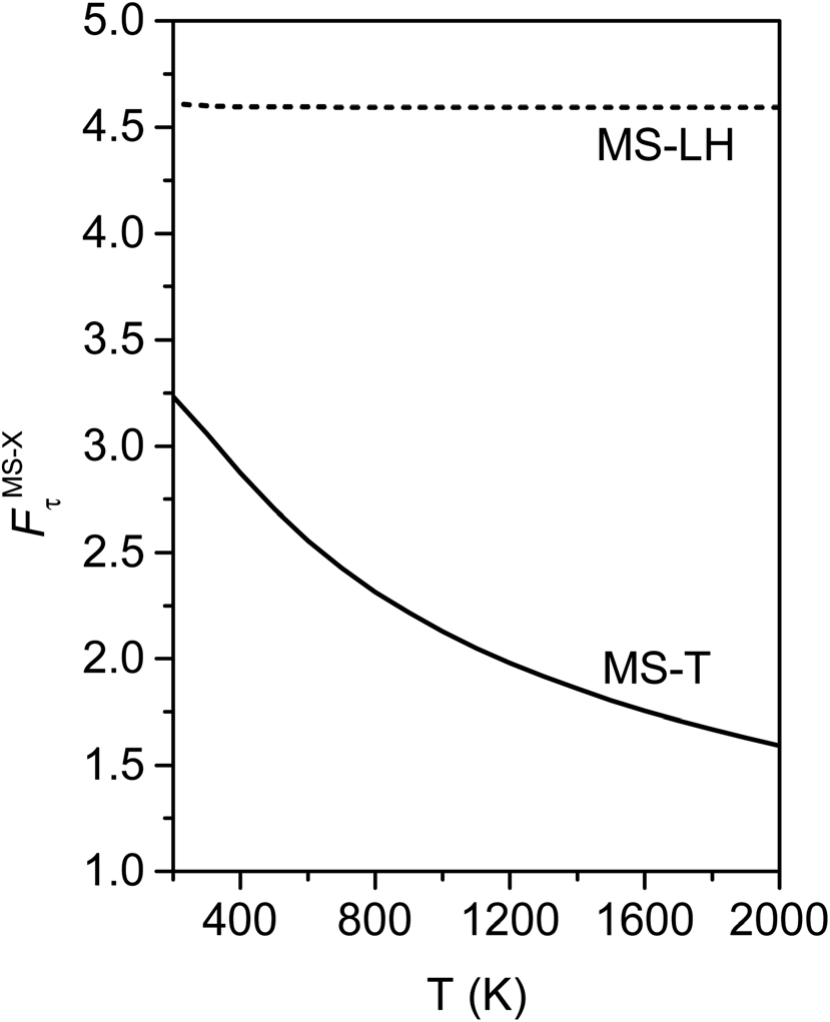
Torsional anharmonicity factors obtained by MS-T and MS-LH methods at 200–2000 K.

Fig. 10 illustrates the torsional anharmonicity factors calculated by the MS-T and MS-LH methods. Unlike the FR or HR treatment, the MS-T method involves no torsion assignment. The MS-T torsional anharmonicity factor ranges from 3.2 at 200 K to 1.5 at 2000 K. The torsional anharmonicity factor obtained by MS(LH) is nearly temperature-independent with a value of 4.6. By comparing the MS-T, FR, and HR results, it is seen that the FR and HR factors by assigning the torsion to *ω*_3_ is the closest to the MS-T result, among the three different assignments. Therefore we go forward with this choice.

### 3.4 Remaining anharmonicity

As explained above, in the present work, we specifically treated torsional anharmonicity of the transition state and the double-minimum mode of the reactant, and the remaining anharmonic effects were included by the quasiharmonic approximation, which is parameterized to give a reasonable estimate of the anharmonic zero point energy, which is dominated by high-frequency modes and is very important at low temperatures; however, this treatment is not reliable for individual low-frequency modes. Therefore one should be concerned about the accuracy of the treatment of nontorsional low-frequency modes because they can make a significant contribution to the entropy at high temperatures. Although the quasiharmonic approximation is not expected to provide a good account of low-frequency anharmonicity at high temperatures, it is expected that some of the vibrational anharmonicity may cancel out in the rate constant calculation. Recently, Jasper *et al.*^73^ estimated a correction factor for anharmonicity by classical Monte Carlo phase space integrals. They estimated, based on these integrals, that when torsional effects are separately estimated (as was done in the present work), the cumulative effect of the remaining vibrational anharmonicity on the partition functions can be estimated as 1.03^*n*_vib_^ at 2500 K (and less at lower temperatures) where *n*_vib_ is the number of vibrational modes. Based on this, one can estimate the overall vibrational anharmonic correction factor of a given reaction. In the present work, the transition state has 45 bound vibrational modes, and the reactants have 40 vibrational modes (39 for cyclopentane and 1 for hydroxyl), so the anharmonicity correction factor at 2500 K can be estimated as
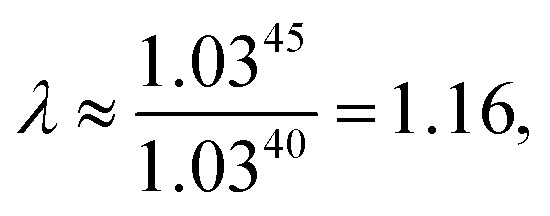


In comparison, our quasiharmonic correction factor at 2500 K is
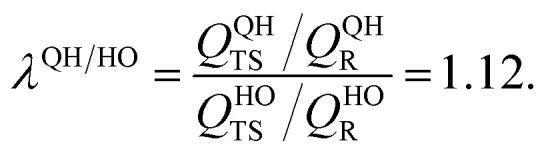


Every reaction has its own special characteristics, and one cannot rely on a cancellation of errors in any specific reaction, so we probably should not put too much stock on the agreement of these two values or on the fact that both methods imply a reasonably small effect of the remaining anharmonicity, even at high temperatures; nevertheless, these estimates are encouraging.

### 3.5 Kinetics

**Fig. 11:**
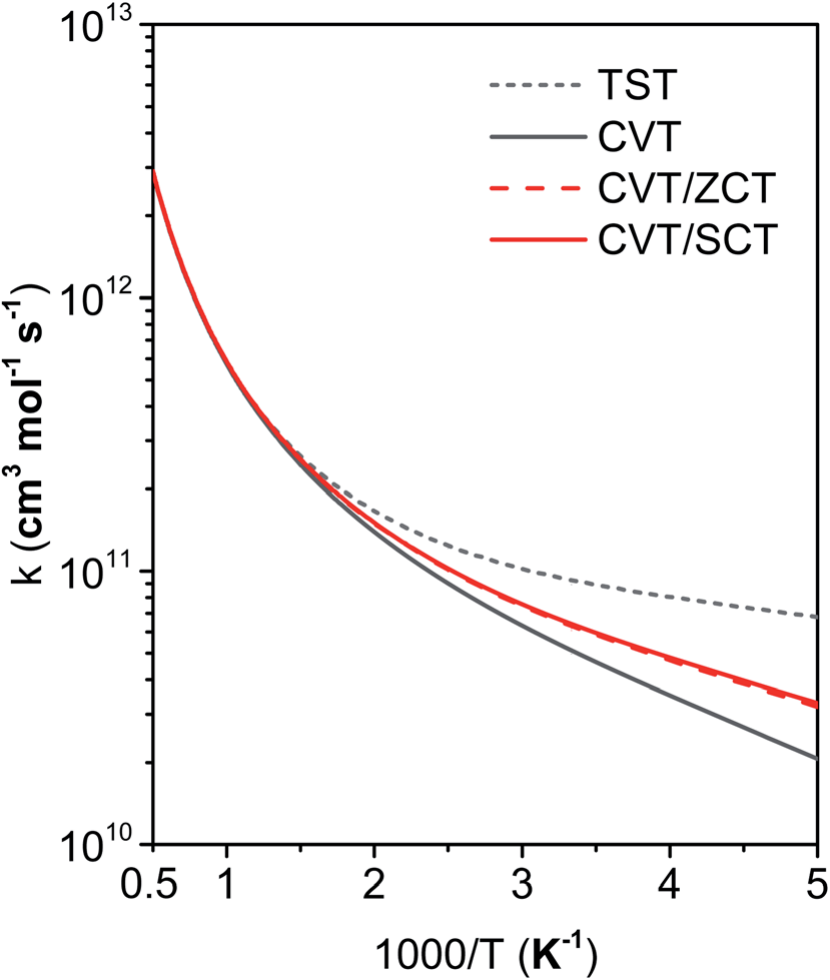
Forward rate constants of cyclopentane reaction with OH radical at 200–2000 K.

Fig. 11 compares the rate constants obtained by TST, CVT, CVT/ZCT and CVT/SCT (the rate constants are tabulated in the ESI†).

#### Variational effect

**Fig. 12:**
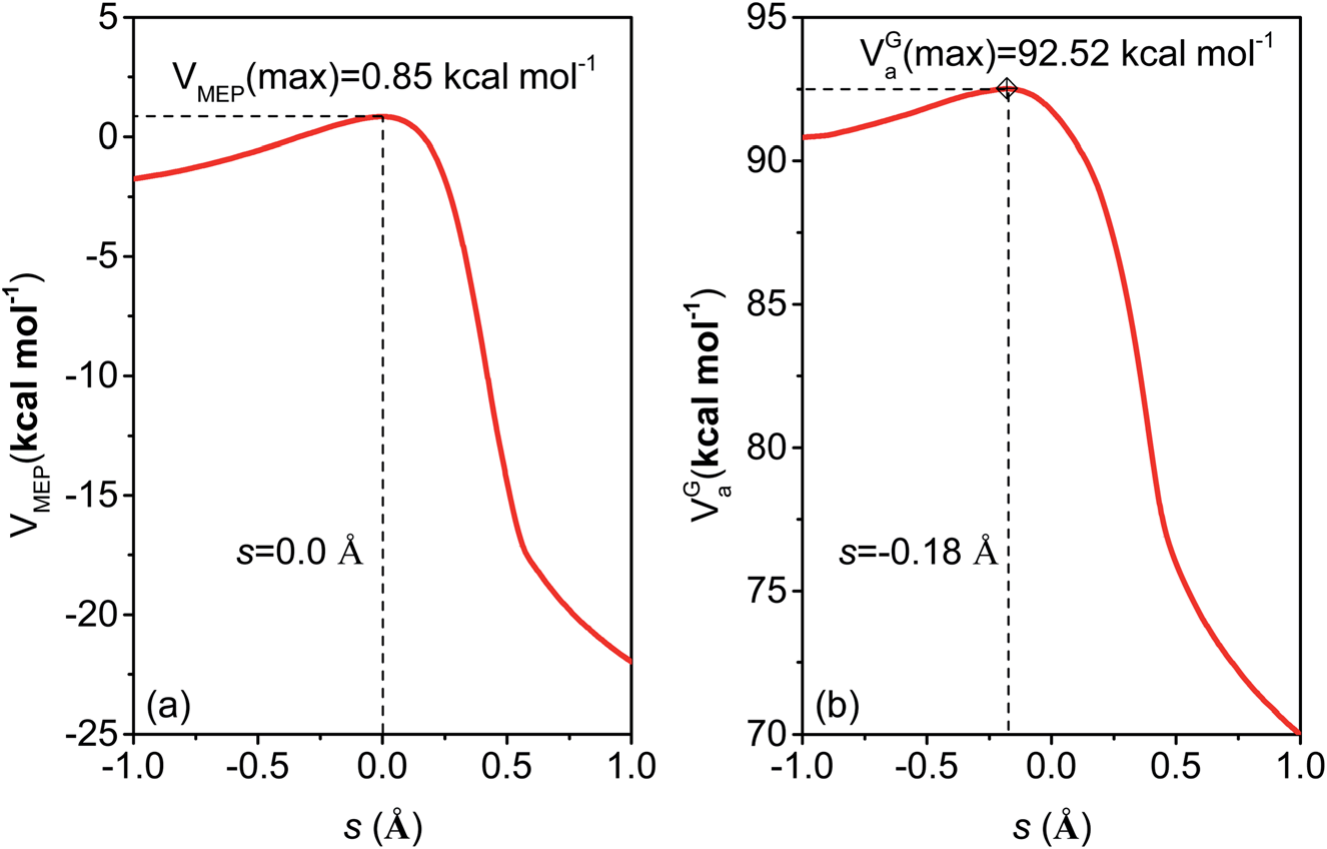
M06-2X/MG3S calculations of (a) *V*_MEP_ and (b) vibrationally adiabatic ground-state potential, *V*^G^_a_, as functions of the reaction coordinate *s*, which is the signed distance from the saddle point along the MEP in isoinertial coordinates scaled to a reduced mass of 1 amu.

The difference of the CVT and TST rate constants is the variational effect, *i.e.*, the effect of taking the transition state as the dividing surface with the highest free energy of activation rather than as the conventional transition state through the saddle point. The figure shows that the variational effect is negligible above 700 K but becomes significant at lower temperatures. For instance, the CVT rate constant at 200 K is smaller than the TST rate constant by a factor of 3.3. To understand the source of this effect, we examine the potential energy curve and the low-temperature limit of free energy of activation profile along the MEP; these are shown in Fig. 12, where we note that the vibrationally adiabatic ground-state curve defined in eqn (3) also serves (within an additive constant) as the generalized free energy of activation profile at 0 K.^50,74^ The low-temperature limit of the dynamical bottleneck is at the maximum of the *V*^G^_a_ curve, and the figure shows this is located 0.18 Å before the transition structure, and this maximum is 0.75 kcal mol^−1^ higher than the value of *V*^G^_a_ at the saddle point. The reason for this is the breaking C–H bond's vibrational frequency, which is decreasing in the transition state region. The frequency of the bond being broken is 3046 cm^−1^ at reactants, 2432 cm^−1^ at *s* = −0.18 Å, and 1658 cm^−1^ at the saddle point. This decrease of the vibrational frequency as one moves along the reaction path is a common occurrence in hydrogen-atom transfer reactions.^74^

#### Tunneling

The CVT/SCT rate constant is slightly larger than the CVT result at low temperatures. For example, the CVT/SCT rate constant at 200 K is larger than the CVT result by a factor of 1.6. The tunneling effects calculated by ZCT and SCT are almost identical. This is because the barrier occurs early along the reaction path (as expected by the Hammond postulate^75,76^) before the system reaches the region where the reaction path curves toward products.

#### Anharmonicity and comparison to experiment

**Fig. 13:**
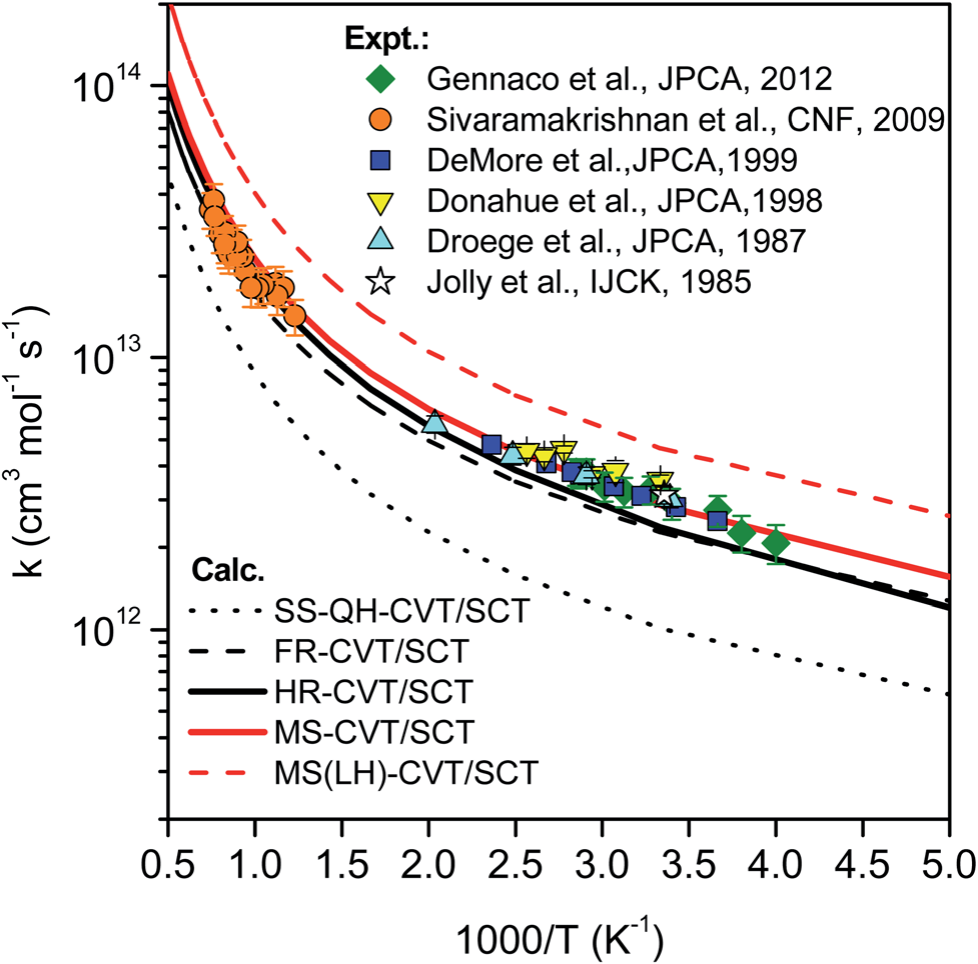
Comparison of rate constants determined in this work by SS-HO-CVT/SCT, SS-FR-CVT/SCT, SS-HR-CVT/SCT, MS-CVT/SCT and MS(LH)-CVT/SCT kinetics theories with the measurements performed by Jolly *et al.*,^19^ Droege *et al.*,^20^ Donahue *et al.*,^23^ DeMore *et al.*,^21^ Sivaramakrishnan *et al.*^25^ and Gennaco *et al.*^24^ The normal mode *ω*_3_ of the transition state is replaced by the torsion in SS-FR-CVT/SCT and SS-HR-CVT/SCT calculations.

Based on the CVT/SCT rate constants, we consider the anharmonic effect by various treatments. Note that the quartic anharmonicity in cyclopentane is incorporated into all calculated rates so the comparisons show the effect of different treatments of the torsional anharmonicity of the transition state. Calculations with five different treatments of the torsional anharmonicity are compared in Fig. 13 to the experimental results, which are available for 233–1347 K.^19–25^

In the first treatment, we only consider the single-structural quasiharmonic oscillator (SS-QH) approximation for the rate constants. The SS-QH-CVT/SCT method underestimates the experimental data by a factor of about 2^1^/2–3 at 250–500 K and a factor of about 2–2^1^/2 at 850–1350 K. Treating the torsion by either the single-structure FR or the single-structure HR approximation improves the predictions; however, as discussed above, the SS-FR-CVT/SCT or SS-HR-CVT/SCT results depend on the torsional mode assignment. A detailed comparison of FR and TES results with experimental data is included in ESI,† which shows that treating the *ω*_3_ mode as the torsion gives better agreement with the experimental measurements for both the FR and HR treatments. When experimental results are not available, one might make a different selection with less accuracy. For large flexible molecules with many torsions, the arbitrariness can be compounded, and the independent-torsion approximation can introduce additional errors. Therefore we do not recommend these independent-torsion single-structure methods in general.

Fig. 13 also shows theoretical results employing the MS-CVT/SCT method, and this gives the best agreement with the experimental results over the entire temperature range. This is especially encouraging because the MS method does not require troublesome torsional assignments, tedious scans, or expensive optimization of torsional barriers.

#### Analytic fits

The temperature dependence of the MS-CVT/SCT rate constants at 250–2000 K was fitted to a four-parameter function:^77^16
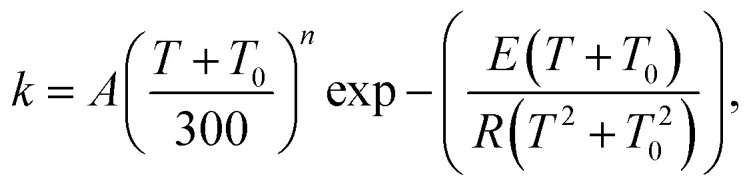
where *A* = 3.37 × 10^11^ cm^3^ mol^−1^ s^−1^, *T*_0_ = 311.8 K, *n* = 2.82, and *E* = −0.11 kcal mol^−1^. We also provide (with the same units) the three-parameter modified Arrhenius form, which is the form preferred by many standard mechanistic programs:17
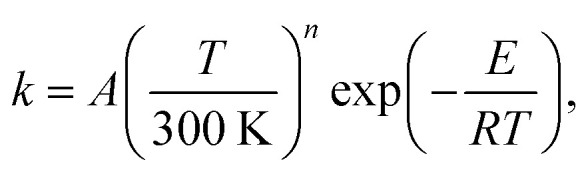
where *A* = 6.30 × 10^11^ cm^3^ mol^−1^ s^−1^, *n* = 2.60, and *E* = −0.94 kcal mol^−1^. To convert the fits to cm^3^ per molecule per s one must divide by Avogadro's number.

## 4. Conclusions

The rate constant for H abstraction from cyclopentane by OH radical at 200–2000 K has been calculated by direct dynamics using multi-structural canonical variational transition state theory including multi-dimensional small-curvature tunneling. An accurate and efficient electronic structure method, M06-2X/MG3S, is identified from among 36 combinations of exchange–correlation functionals and basis set by comparing the reaction energy and forward and reverse barrier heights for CCSD(T)-F12a/jun-cc-pVTZ calculations. The mean unsigned deviation of M06-2X/MG3S from the benchmark coupled cluster calculations is only 0.22 kcal mol^−1^. A quadratic–quartic potential is adopted for the bending anharmonicity in cyclopentane. For the transition state, the torsional anharmonicity at the abstraction site has been treated by FR, HR, MS-T, and MS(LH) methods. The performances of the FR and HR approximations depend on the assignment of torsion to a normal mode, which is subjective. The rate constant determined by the MS-T method, which avoids this problem by using internal coordinates for the torsion, shows excellent agreement with the experimental data over the entire temperature range of 200–2000 K. Based on our observations, we recommend the MS-T method for reliable anharmonicity corrections. Because of the good agreement of the calculations with experiment, they can be used for rate constants at temperatures where experiments are not available. Furthermore, the present paper validates the present theoretical methods for reactions involving anharmonic ring systems so that these methods may be applied with more confidence to the many reactions of ring molecules where experiments are not available.

## Conflicts of interest

There are no conflicts to declare.

## Supplementary Material

SC-011-C9SC05632G-s001
