# Supplementary material for: Anharmonic kinetics of the cyclopentane reaction with hydroxyl radical
Source: Chem Sci. 2020 Jan 25;11(9):2511–23. doi: 10.1039/c9sc05632g (PMC8157450; doi:10.1039/c9sc05632g)
Supplement: SC-011-C9SC05632G-s001 [file SC-011-C9SC05632G-s001.pdf]

Electronic Supplementary Material (ESI) for Chemical Science.
This journal is © The Royal Society of Chemistry 2020

Anharmonic Kinetics of the Cyclopentane Reaction with Hydroxyl Radical

Junjun Wu[a,b], Lu Gem Gao[b,c], Wei Ren*[b], Donald G. Truhlar*[a]

[a] Department of Mechanical and Automation Engineering, Shenzhen Research Institute, The Chinese University of Hong Kong, New Territories, Hong Kong, China

[b] Department of Chemistry, Chemical Theory Center, and Supercomputing Institute, University of Minnesota, Minneapolis, USA

[c] Center for Combustion Energy, Department of Energy and Power Engineering, and Key Laboratory for Thermal Science and Power Engineering of Ministry of Education, Tsinghua University, Beijing, China

Email: renwei@mae.cuhk.edu.hk; truhlar@umn.edu

Table of Contents

Quadratic-quartic potential details ········ S-3

Figure S1. Labeled geometries of half-chair cyclopentane ········ S-6

Figure S2. Bond lengths and bending angles of cyclopentane and cyclopentyl ········ S-6

Table S1. The C−H Bond dissociation energies of cyclopentane ········ S-7

Table S2. Electronic structure calculations with M08-HX/MG3S geometries ········ S-8

Table S3. Electronic structure calculations with M06-2X/MG3S geometries ········ S-9

Figure S3. 1D torsional potentials of C−O torsion ········ S-10

Internal Coordinates for MS-T Calculation ········ S-10

Table S4. The anharmonic scaling factors at 200-2000 K ········ S-11

Table S5. Rate constants at 200-2000 K by various kinetics theories ········ S-11

Figure S4. SS-FR/HR-CVT/SCT rate constants compared to experiment measurements·······S-12

References···S-12

Cartesian coordinates of the M06-2X/MG3S optimized structures·······································S-13

1. Details of Quadratic-Quartic Potential

Assume a quadratic-quartic potential for the bending mode (#39) in cyclopentane, where the half-chair (HC) is the mimima and the envelope (envo) is the saddle point. So the potential is expressed as:

$$V = \frac{1}{2}kq_m{}^2 + Aq_m{}^4, \tag{1}$$

The first derivative is:

$$\frac{\partial V}{\partial q_m} = kq_m + 4Aq_m{}^3, \tag{2}$$

The second derivative is:

$$\frac{\partial^2 V}{\partial q_m{}^2} = k + 12Aq_m{}^2, \tag{3}$$

Given the distance between the envo and HC is q_{HC}, at the stationary points, $\frac{\partial V}{\partial q_m} = 0$, therefore:

$$\left.\frac{\partial V}{\partial q_m}\right|_{q_m = 0} = 0, \text{ and } \left.\frac{\partial V}{\partial q_m}\right|_{q_m = \pm q_{\mathrm{HC}}} = 0, \tag{4}$$

So substitute equation (4) into equation (2), we can get:

$$\frac{\partial V}{\partial q_m} = q_{\mathrm{HC}}\left(k + 4Aq_{\mathrm{HC}}{}^2\right) = 0, \tag{5}$$

The solution to equation (5) is: $q_{\mathrm{HC}} = \frac{1}{2}\sqrt{\frac{-k}{A}}$, (6)

Take (6) into (3) we can thus get:

$$\left.\frac{\partial^2 V}{\partial q_m{}^2}\right|_{q_m = 0} = k, \tag{7a}$$

$$\left.\frac{\partial^2 V}{\partial {q_m}^2}\right|_{q_m = q_{\mathrm{HC}}} = k + 12A \cdot \frac{-k}{4A} = -2k, \tag{7b}$$

So the frequencies of Mode #39 of envo and HC conformers are:

$$\omega_{\mathrm{envo}} = \sqrt{\frac{\partial V^2}{\partial {q_e}^2} \bigg/ \mu} = \sqrt{k/\mu}, \tag{8a}$$

$$\omega_{\mathrm{HC}} = \sqrt{\frac{\partial V^2}{\partial {q_e}^2} \bigg/ \mu} = \sqrt{-2k/\mu}, \tag{8b}$$

We can thus know that the frequency #39 of HC is $\sqrt{2}$ larger than that of envo in magnitude.

From our Gaussian calculation, we know that $\omega_{\mathrm{envo}} = 10.15i$, and $\omega_{\mathrm{HC}} = 14.20$, so:

$$\left|\frac{\omega_{\mathrm{HC}}}{\omega_{\mathrm{envo}}}\right| = 1.40 \approx \sqrt{2}.$$

We can thus find that k=-1.53×10^{-4} J/m^2 from Equation (8a) and k=-1.51×10^{-4} J/m^2 from Equation (8b), and We take the average value as $k = -1.52\times10^{-4}$ J/m^2.

Now let's turn back to Equation (6), the coefficient A for the quartic term is available:

$$A = \frac{-k}{4{q_{\mathrm{HC}}}^2}, \tag{9}$$

Before getting q_{HC}, we implemeted rotational operation to make sure the half-chair and envelope structures in the maxium superpostion. According to Chen's work, the rotational matrix is:

$$R = \begin{pmatrix} \cos\alpha & -\sin\alpha & 0 \\ \sin\alpha & \cos\alpha & 0 \\ 0 & 0 & 1 \end{pmatrix} \begin{pmatrix} \cos\beta & 0 & -\sin\beta \\ 0 & 1 & 0 \\ \sin\beta & 0 & \cos\beta \end{pmatrix} \begin{pmatrix} 1 & 0 & 0 \\ 0 & \cos\gamma & -\sin\gamma \\ 0 & \sin\gamma & \cos\gamma \end{pmatrix} \tag{10}$$

After several interaction steps, the structure of HC is finalized and pending below.

Intial structure of HC				Final structure of HC			
C	0.341116	0.683759	-1.02228	C	0.495742	1.164801	-0.16255
C	0	1.235171	0.366581	C	-0.07324	0.593749	1.141108
C	0	0	1.299694	C	-0.2119	-0.92682	0.886176
C	0	-1.23517	0.366581	C	-0.04629	-1.11657	-0.64121
C	-0.34112	-0.68376	-1.02228	C	-0.1624	0.293188	-1.23151
H	0.013459	1.334973	-1.83161	H	0.297337	2.228909	-0.28407
H	1.421668	0.550416	-1.11872	H	1.57895	1.022189	-0.19414
H	-0.99534	1.681397	0.340081	H	-1.05574	1.031265	1.324894
H	0.690444	2.009859	0.695971	H	0.545774	0.819126	2.00793
H	0.873925	-0.00537	1.949393	H	0.544416	-1.49014	1.430731
H	-0.87393	0.005371	1.949393	H	-1.18006	-1.29011	1.227595
H	0.995335	-1.6814	0.340081	H	0.944851	-1.51629	-0.86114
H	-0.69044	-2.00986	0.695971	H	-0.77271	-1.81173	-1.05886
H	-0.01346	-1.33497	-1.83161	H	0.299902	0.38336	-2.21365
H	-1.42167	-0.55042	-1.11872	H	-1.21416	0.57335	-1.33143

* the maximum residual error is 2×10^{-16} Å.

So we can compute the path length by directly comparing their mass-scaled coordinates.

$$\Delta q = \left| q^{\mathrm{E}} - q^{\mathrm{HC}} \right| = \sqrt{\sum \left(q_m{}^{\mathrm{E}} - q_m{}^{\mathrm{HC}} \right)^2} = 0.76 \text{ Å},$$

Therefore, we can obtain $A = 6.58\times10^{15}$ J/m^4.

2. C-C Bond Dissociation Energies by M06-2X/MG3S

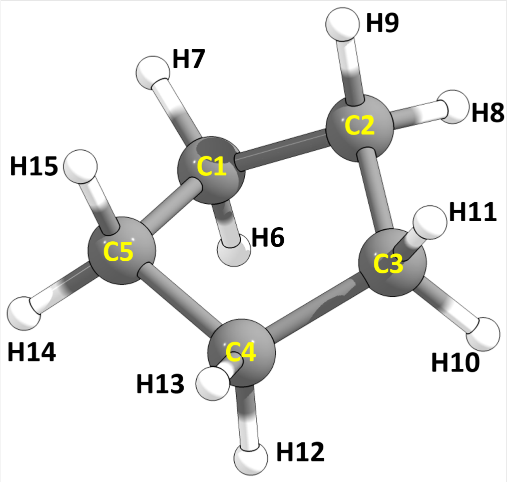

Figure S1. The geometry and atom numbering of cyclopentane (cycloC5).

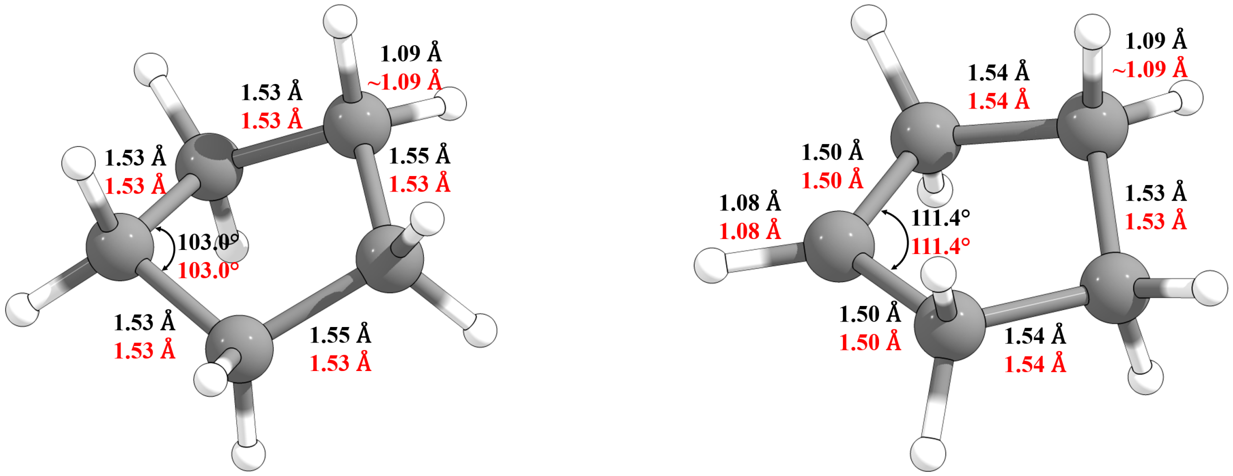

Figure S2. Detailed geometric parameters of cyclopentane and cyclopentyl optimized by M06-2X/MG3S (in black) and M08-HX/MG3S (in red).

Table S1. Single-point energies of cyclopentane (cycloC5), H atom, and cyclopentyl radicals (R*i*) obtained by abstractions at various bonds, and the derived bond dissociation energies (BDEs). The BDE is calculated by BDE = E(Ri) + E(H) – E(cycloC5). All these calculations are carried out by M06-2X/MG3S.

Species	Energies (Hartree)	Bond	BDE (kcal·mol^{-1})
cycloC5	-196.513612		
H	-0.498134		
R1	-195.852098	C1−H6	102.52
R2	-195.852098	C1−H7	102.52
R3	-195.852098	C2−H8	102.52
R4	-195.852098	C2−H9	102.52
R5	-195.852098	C3−H10	102.52
R6	-195.852098	C3-H11	102.52
R7	-195.852098	C4−H12	102.52
R8	-195.852098	C4−H13	102.52
R9	-195.852098	C5-H14	102.52
R10	-195.852098	C5−H15	102.52

3. Electronic Structure Calculations

Table S2. Forward ($V_f^{\neq}$) and reverse ($V_r^{\neq}$) barrier height, energy of reaction (ΔV), mean signed deviation (MSD) and mean unsigned deviation MUEs from the coupled cluster benchmark for cyclopentane + OH (the unit is kcal/mol). These are all single-point energy calculations based on the structures optimized by M08-HX/MG3S.

DFT	Basis Set	$V_f^{\neq}$	$V_r^{\neq}$	ΔV	MSD	MUD
**CCSD(T)-F12a/jun-cc-PVTZ**		**1.27**	**22.65**	**-21.38**	**-**	**-**
B3LYP	MG3S	-2.29	19.86	-22.15	-2.37	2.37
B3LYP	jun-cc-pVTZ	-2.16	19.99	-22.15	-2.29	2.29
B3LYP	jul-cc-pVTZ	-2.17	19.96	-22.12	-2.29	2.29
B3LYP	aug-cc-pVTZ	-2.2	19.99	-22.19	-2.31	2.31
M05-2X	MG3S	1.05	22.96	-21.91	-0.15	0.35
M05-2X	jun-cc-pVTZ	0.94	23.16	-22.22	-0.22	0.56
M05-2X	jul-cc-pVTZ	0.9	23.12	-22.22	-0.25	0.56
M05-2X	aug-cc-pVTZ	0.83	23.11	-22.27	-0.29	0.6
**M06-2X**	**MG3S**	**0.87**	**22.35**	**-21.48**	**-0.27**	**0.27**
M06-2X	jun-cc-pVTZ	0.85	22.68	-21.83	-0.28	0.3
M06-2X	jul-cc-pVTZ	0.8	22.63	-21.83	-0.31	0.31
M06-2X	aug-cc-pVTZ	0.75	22.62	-21.87	-0.35	0.35
M08-HX	MG3S	1.74	23.78	-22.04	0.31	0.75
M08-HX	jun-cc-pVTZ	2.02	24.27	-22.25	0.5	1.08
M08-HX	jul-cc-pVTZ	1.95	24.16	-22.22	0.45	1.01
M08-HX	aug-cc-pVTZ	1.87	24.09	-22.22	0.4	0.96
M08-SO	MG3S	0.39	23.55	-23.16	-0.59	1.19
M08-SO	jun-cc-pVTZ	0.89	23.69	-22.8	-0.25	0.95
M08-SO	jul-cc-pVTZ	0.82	23.59	-22.78	-0.3	0.93
M08-SO	aug-cc-pVTZ	0.76	23.55	-22.79	-0.34	0.94
MN15	MG3S	-0.21	23.03	-23.24	-0.99	1.24
MN15	jun-cc-pVTZ	0.05	23.13	-23.08	-0.81	1.13
MN15	jul-cc-pVTZ	-0.05	23.01	-23.06	-0.88	1.12
MN15	aug-cc-pVTZ	-0.12	22.99	-23.11	-0.93	1.15
MN15-L	MG3S	0.31	23.22	-22.91	-0.64	1.02
MN15-L	jun-cc-pVTZ	0.38	23.11	-22.73	-0.59	0.9
MN15-L	jul-cc-pVTZ	0.26	22.96	-22.7	-0.67	0.88
MN15-L	aug-cc-pVTZ	0.24	23	-22.76	-0.69	0.92
MP1WP	MG3S	2.38	22.58	-20.2	0.74	0.79
MP1WP	jun-cc-pVTZ	2.64	22.82	-20.18	0.91	0.91
MP1WP	jul-cc-pVTZ	2.64	22.78	-20.14	0.91	0.91
MP1WP	aug-cc-pVTZ	2.59	22.77	-20.18	0.88	0.88
ωB97X-D	MG3S	-1.7	19.91	-21.61	-1.98	1.98
ωB97X-D	jun-cc-pVTZ	-1.35	19.97	-21.32	-1.75	1.79
ωB97X-D	jul-cc-pVTZ	-1.38	19.92	-21.31	-1.77	1.82
ωB97X-D	aug-cc-pVTZ	-1.42	19.92	-21.34	-1.79	1.82

Table S3. Forward ($V_f^{\neq}$) and reverse ($V_r^{\neq}$) barrier height, energy of reaction (ΔV), mean signed deviation (MSD) and mean unsigned deviation MUEs from the coupled cluster benchmark for cyclopentane + OH (the unit is kcal/mol). These are all single-point energy calculations based on the structures optimized by M06-2X/MG3S.

DFT	Basis Set	$V_f^{\neq}$	$V_r^{\neq}$	ΔV	MSD	MUD
B3LYP	MG3S	-2.32	19.82	-22.14	-2.34	2.34
B3LYP	jun-cc-pVTZ	-2.19	19.95	-22.14	-2.25	2.25
B3LYP	jul-cc-pVTZ	-2.2	19.92	-22.11	-2.25	2.25
B3LYP	aug-cc-pVTZ	-2.22	19.95	-22.17	-2.27	2.27
M05-2X	MG3S	1.03	22.9	-21.87	-0.1	0.31
M05-2X	jun-cc-pVTZ	0.94	23.12	-22.18	-0.16	0.52
M05-2X	jul-cc-pVTZ	0.89	23.08	-22.19	-0.2	0.52
M05-2X	aug-cc-pVTZ	0.83	23.07	-22.24	-0.24	0.56
**M06-2X**	**MG3S**	**0.85**	**22.32**	**-21.47**	**-0.22**	**0.22**
M06-2X	jun-cc-pVTZ	0.84	22.66	-21.82	-0.23	0.28
M06-2X	jul-cc-pVTZ	0.8	22.61	-21.82	-0.26	0.27
M06-2X	aug-cc-pVTZ	0.74	22.6	-21.86	-0.3	0.3
M08-HX	MG3S	1.75	23.8	-22.05	0.38	0.81
M08-HX	jun-cc-pVTZ	2.05	24.3	-22.25	0.58	1.14
M08-HX	jul-cc-pVTZ	1.98	24.19	-22.22	0.53	1.07
M08-HX	aug-cc-pVTZ	1.9	24.12	-22.22	0.48	1.02
M08-SO	MG3S	0.47	23.66	-23.18	-0.47	1.19
M08-SO	jun-cc-pVTZ	0.96	23.79	-22.82	-0.15	0.95
M08-SO	jul-cc-pVTZ	0.96	23.79	-22.82	-0.15	0.95
M08-SO	aug-cc-pVTZ	0.83	23.64	-22.81	-0.24	0.94
MN15	MG3S	-0.23	23	-23.23	-0.94	1.22
MN15	jun-cc-pVTZ	0.02	23.09	-23.07	-0.78	1.11
MN15	jul-cc-pVTZ	-0.07	22.98	-23.05	-0.84	1.1
MN15	aug-cc-pVTZ	-0.15	22.95	-23.10	-0.89	1.13
MN15-L	MG3S	0.36	23.37	-23.01	-0.55	1.07
MN15-L	jun-cc-pVTZ	0.43	23.25	-22.83	-0.51	0.95
MN15-L	jul-cc-pVTZ	0.31	23.10	-22.80	-0.59	0.93
MN15-L	aug-cc-pVTZ	0.28	23.14	-22.86	-0.6	0.97
MP1WP	MG3S	2.23	22.37	-20.14	0.7	0.84
MP1WP	jun-cc-pVTZ	2.49	22.61	-20.12	0.87	0.87
MP1WP	jul-cc-pVTZ	2.48	22.57	-20.09	0.86	0.88
MP1WP	aug-cc-pVTZ	2.44	22.56	-20.13	0.83	0.85
ωB97X-D	MG3S	-1.73	19.86	-21.59	-1.94	1.94
ωB97X-D	jun-cc-pVTZ	-1.39	19.91	-21.30	-1.72	1.78
ωB97X-D	jul-cc-pVTZ	-1.42	19.87	-21.29	-1.74	1.81
ωB97X-D	aug-cc-pVTZ	-1.46	19.86	-21.32	-1.76	1.82
**CCSD(T)-F12a/jun-cc-pVTZ**		**1.18**	**22.59**	**-21.40**	-	-
**CCSD(T)-F12b/jun-cc-pVTZ**		**1.17**	**22.55**	**-21.38**	-0.02	0.02

4. Torsional Anharmonicity

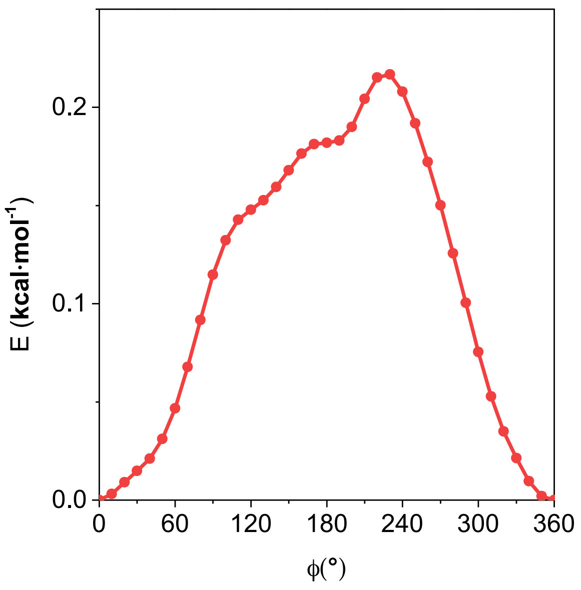

Figure S3. Relaxed torsional potential energy curve for the C–O torsion at the transition state as calculated by M06-2X/MG3S.

MS-T Internal Coordinates for TS: Two Torsions

```
### Stretches=17 ###
1-6 1-7 1-2 2-8 2-9 2-3 3-10 3-11 3-4 4-12 4-13 4-5 5-14 5-15 5-1 16-17 6-16
### Bends=26 ###
6-1-2 6-1-7 6-1-5 7-1-2 5-1-2
8-2-9 8-2-1 8-2-3 9-2-3 1-2-3
10-3-2 10-3-4 10-3-11 11-3-4 2-3-4
12-4-3 12-4-5 12-4-13 13-4-5 3-4-5
14-5-4 14-5-1 14-5-15 15-5-1 1-6-16
6-16-17
### R=Torsions=2 ###
2-1-6-16 1-6-16-17
END
```

MS-T Internal Coordinates for TS: One Torsion

```
### Stretches=17 ###
1-6 1-7 1-2 2-8 2-9 2-3 3-10 3-11 3-4 4-12 4-13 4-5 5-14 5-15 5-1 16-17 1-16
### Bends=27 ###
6-1-2 6-1-7 6-1-5 7-1-2 5-1-2
8-2-9 8-2-1 8-2-3 9-2-3 1-2-3
10-3-2 10-3-4 10-3-11 11-3-4 2-3-4
12-4-3 12-4-5 12-4-13 13-4-5 3-4-5
14-5-4 14-5-1 14-5-15 15-5-1 1-6-16
2-1-16 1-16-17
### Torsions=1 ###
2-1-16-17
END
```

Table S4. The torsional anharmonicity factors calculated by the FR, HR, MS-T and MS-LH methods.

T (K)	F^{FR}			F^{HR}			F^{MS-T}	F^{MS-LH}
	ω_1	ω_2	ω_3	ω_1	ω_2	ω_3		
200	1.86	2.06	2.65	1.75	1.95	2.5	3.233	4.610
300	1.63	1.84	2.43	1.7	1.91	2.53	3.06	4.601
400	1.47	1.66	2.23	1.61	1.82	2.45	2.873	4.597
500	1.34	1.53	2.07	1.52	1.72	2.35	2.704	4.596
600	1.25	1.42	1.94	1.44	1.64	2.24	2.556	4.595
700	1.17	1.33	1.83	1.37	1.56	2.15	2.428	4.594
800	1.1	1.26	1.74	1.3	1.49	2.06	2.315	4.594
900	1.05	1.19	1.65	1.25	1.43	1.98	2.217	4.594
1000	1	1.14	1.58	1.2	1.37	1.91	2.129	4.593
1100	0.95	1.09	1.52	1.16	1.32	1.84	2.05	4.593
1200	0.92	1.05	1.46	1.12	1.28	1.78	1.98	4.593
1300	0.88	1.01	1.41	1.08	1.24	1.73	1.916	4.593
1400	0.85	0.98	1.37	1.05	1.2	1.68	1.858	4.593
1600	0.83	0.94	1.32	0.99	1.13	1.59	1.755	4.593
1700	0.8	0.92	1.29	0.96	1.1	1.55	1.71	4.593
1800	0.78	0.89	1.25	0.94	1.08	1.51	1.668	4.593
1900	0.76	0.87	1.22	0.92	1.05	1.48	1.629	4.593
2000	0.74	0.85	1.19	0.9	1.03	1.45	1.593	4.593

5. Rate Constants

Table S5. Rate constants (in $cm^3 \cdot molecule^{-1} \cdot s^{-1}$) as a function of temperature (in K).

T	TST	CVT	TST/ZCT	CVT/ZCT	TST/SCT	CVT/SCT	QQ/QH[a]	MS-CVT/SCT
200	2.26E-12	6.85E-13	5.32E-13	9.11E-13	5.50E-13	9.42E-13	0.85	2.59E-12
300	2.96E-12	1.65E-12	1.03E-12	1.65E-12	1.05E-12	1.68E-12	0.93	4.77E-12
400	4.02E-12	2.94E-12	1.76E-12	2.60E-12	1.78E-12	2.63E-12	0.99	7.50E-12
500	5.44E-12	4.56E-12	2.76E-12	3.77E-12	2.77E-12	3.79E-12	1.05	1.07E-11
600	7.23E-12	6.53E-12	4.07E-12	5.20E-12	4.08E-12	5.22E-12	1.09	1.46E-11
700	9.45E-12	8.92E-12	5.74E-12	6.94E-12	5.75E-12	6.96E-12	1.13	1.92E-11
800	1.22E-11	1.18E-11	7.82E-12	9.04E-12	7.84E-12	9.06E-12	1.17	2.46E-11
900	1.54E-11	1.51E-11	1.04E-11	1.16E-11	1.04E-11	1.16E-11	1.20	3.09E-11
1000	1.92E-11	1.89E-11	1.34E-11	1.46E-11	1.34E-11	1.46E-11	1.23	3.84E-11
1100	2.36E-11	2.34E-11	1.70E-11	1.81E-11	1.70E-11	1.81E-11	1.26	4.69E-11
1200	2.86E-11	2.85E-11	2.12E-11	2.21E-11	2.12E-11	2.21E-11	1.29	5.66E-11
1300	3.43E-11	3.43E-11	2.60E-11	2.67E-11	2.60E-11	2.67E-11	1.32	6.75E-11
1400	4.07E-11	4.07E-11	3.14E-11	3.19E-11	3.15E-11	3.20E-11	1.34	7.97E-11

1500	4.79E-11	4.79E-11	3.76E-11	3.78E-11	3.76E-11	3.78E-11	1.37	9.32E-11
1600	5.59E-11	5.59E-11	4.45E-11	4.44E-11	4.45E-11	4.44E-11	1.39	1.08E-10
1700	6.47E-11	6.46E-11	5.22E-11	5.17E-11	5.22E-11	5.17E-11	1.41	1.25E-10
1800	7.43E-11	7.42E-11	6.06E-11	5.98E-11	6.06E-11	5.98E-11	1.43	1.42E-10
1900	8.48E-11	8.47E-11	6.99E-11	6.87E-11	6.99E-11	6.87E-11	1.45	1.62E-10
2000	9.62E-11	9.60E-11	8.00E-11	7.84E-11	8.01E-11	7.84E-11	1.47	1.83E-10

[a]QQ/QH is the quadratic-quartic anharmonicity correcting factor. The MS-CVT/SCT rate constants are the products of CVT/SCT rate constants and QQ/QH and MS-T factors. The MS-T factor is in Table S4.

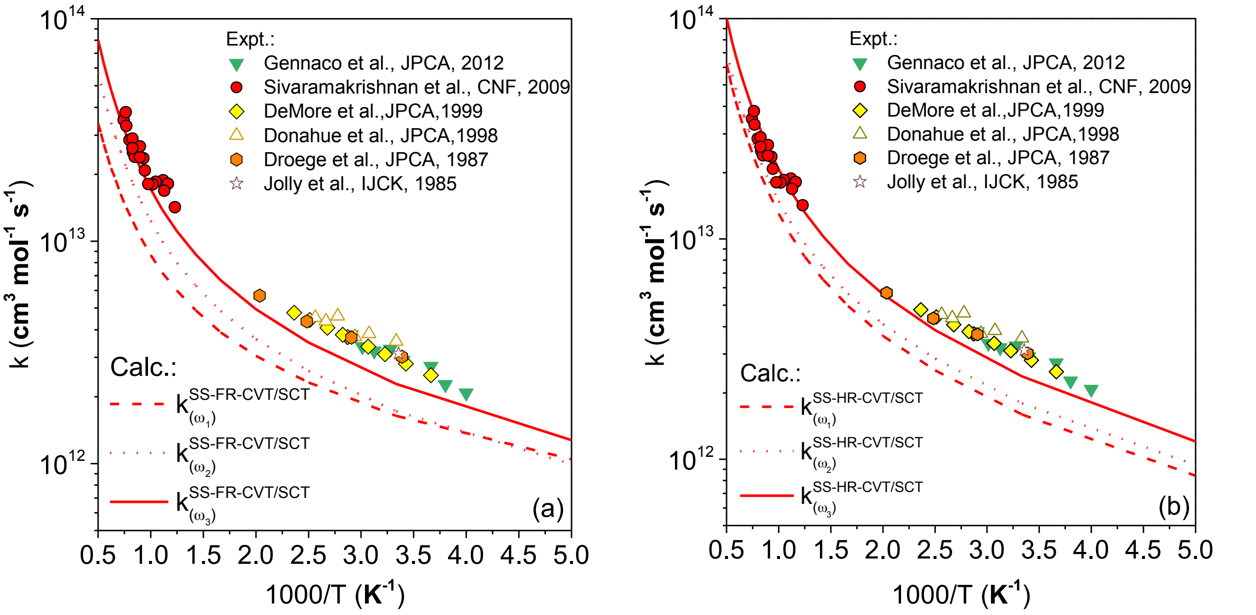

Figure S4. CVT/SCT rate constants by different anharmonic treatments: (a) single structural FR, (b) single structural HR. In the notation of rate constant, the headnote indicates the kinetic theory; the footnote indicate the torsion considered. The normal modes ω_i replaced by torsions are shown in the brackets. The measurements were performed by Jolly et al.[1], Droege et al.[2], Donahue et al.[3], DeMore et al.[4], Sivaramakrishnan et al.[5] and Gennaco et al.[6].

References

1 G. S. Jolly, G. Paraskevopoulos and D. L. Singleton, *Int J Chem Kinet*, 1985, **17**, 1–10.

2 A. T. Droege and F. P. Tully, *J Phys Chem*, 1987, **91**, 1222–1225.

3 N. M. Donahue, J. G. Anderson and K. L. Demerjian, *J Phys Chem A*, 1998, **102**, 3121–3126.

4 W. B. DeMore and K. D. Bayes, *J Phys Chem A*, 1999, **103**, 2649–2654.

5 R. Sivaramakrishnan and J. V. Michael, *Combust Flame*, 2009, **156**, 1126–1134.

6 M. A. Gennaco, Y. W. Huang, R. A. Hannun and T. J. Dransfield, *J Phys Chem A*, 2012, **116**, 12438–12443.

6. Optimized Cartesian coordinates (in Å) of reactants, products and the transition states

Cyclopentane (half-chair)

C	0.34111600	0.68375900	-1.02228000
C	0.00000000	1.23517100	0.36658100
C	0.00000000	0.00000000	1.29969400
C	0.00000000	-1.23517100	0.36658100
C	-0.34111600	-0.68375900	-1.02228000
H	0.01345900	1.33497300	-1.83161200
H	1.42166800	0.55041600	-1.11872400
H	-0.99533500	1.68139700	0.34008100
H	0.69044400	2.00985900	0.69597100
H	0.87392500	-0.00537100	1.94939300
H	-0.87392500	0.00537100	1.94939300
H	0.99533500	-1.68139700	0.34008100
H	-0.69044400	-2.00985900	0.69597100
H	-0.01345900	-1.33497300	-1.83161200
H	-1.42166800	-0.55041600	-1.11872400

Cyclopentane (envelope)

C	-0.50876600	-1.16835600	0.00000000
C	0.12689100	-0.45205700	1.19217600
C	0.12689100	1.03034200	0.77600000
C	0.12689100	1.03034200	-0.77600000
C	0.12689100	-0.45205700	-1.19217600
H	-0.33739100	-2.24409500	0.00000000
H	-1.58925100	-1.00107300	0.00000000
H	1.15249600	-0.80664400	1.31590900
H	-0.39496800	-0.62631800	2.13201400
H	-0.76968000	1.52341000	1.15135300
H	0.97908200	1.56749400	1.18875100
H	-0.76968000	1.52341000	-1.15135300
H	0.97908200	1.56749400	-1.18875100
H	-0.39496800	-0.62631800	-2.13201400
H	1.15249600	-0.80664400	-1.31590900

OH

O	0.00000000	0.00000000	0.10787600
H	0.00000000	0.00000000	-0.86301100

TS1 (Torsional Conformer # 1)

C	-0.38411700	0.32969300	0.9019280
C	0.22766600	-1.06120800	0.7678450
C	0.91911600	-0.99599400	-0.5941130
C	1.57678800	0.38768100	-0.5720190

C	0.56204700	1.29247300	0.1618930
H	-1.36477500	0.33365500	0.3182750
H	-0.64066400	0.62673100	1.9172070
H	-0.50696000	-1.86186400	0.8487570
H	0.97255200	-1.21451600	1.5544610
H	1.62948200	-1.80449400	-0.7592710
H	0.16292800	-1.03825100	-1.3808700
H	2.50659100	0.33521700	-0.0027840
H	1.82358500	0.75719600	-1.5654430
H	1.05542100	1.97553000	0.8525850
H	-0.00001400	1.90523200	-0.5425100
O	-2.51011300	0.10232500	-0.6431900
H	-2.96624600	-0.54890100	-0.0880860

TS2 (Torsional Conformer # 2)

C	-0.37292700	0.19100800	0.98972500
C	0.34445300	-1.12535200	0.72241500
C	0.90591800	-0.91923700	-0.68437700
C	1.42828100	0.52373600	-0.64694400
C	0.47078100	1.27889500	0.30843100
H	-1.39067500	0.11524400	0.46735500
H	-0.61737000	0.38830500	2.03212200
H	-0.31218700	-1.98932300	0.80909500
H	1.16436100	-1.25023200	1.43660700
H	1.67547400	-1.64032900	-0.95470500
H	0.09210900	-1.01202200	-1.40678800
H	2.43897200	0.53260500	-0.23730900
H	1.47988600	0.97859200	-1.63456600
H	1.02803300	1.85379500	1.04790100
H	-0.15720100	1.99463000	-0.22287900
O	-2.49097500	-0.03453400	-0.53818800
H	-2.13263200	0.61070600	-1.16684000

Cyclopentyl

C	0.72896600	-0.98015100	0.23596600
C	1.22827500	0.42859100	-0.12680500
C	-0.00002900	1.27036200	-0.00019500
C	-1.22823800	0.42858500	0.12700600
C	-0.72901200	-0.98006200	-0.23610700
H	1.32469300	-1.77501500	-0.20952500
H	0.76045500	-1.10754800	1.32022000
H	1.61201300	0.44005600	-1.15623100
H	2.05145200	0.76582100	0.50689400
H	0.00003100	2.34915900	0.00017100
H	-1.61131600	0.43987700	1.15669800
H	-2.05184200	0.76583500	-0.50611900
H	-1.32477300	-1.77505100	0.20911300
H	-0.76048200	-1.10709000	-1.32041100

H_2O

O	0.00000000	0.00000000	0.11623000
H	0.00000000	0.76158900	-0.46492000
H	0.00000000	-0.76158900	-0.46492000
